# Polyphenol oxidase and enzymatic browning in apricot (*Prunus armeniaca* L.): Effect on phenolic composition and deduction of main substrates

**DOI:** 10.1016/j.crfs.2021.12.015

**Published:** 2022-01-04

**Authors:** Ala eddine Derardja, Matthias Pretzler, Ioannis Kampatsikas, Milena Radovic, Anna Fabisikova, Martin Zehl, Malika Barkat, Annette Rompel

**Affiliations:** a Universität Wien, Fakultät für Chemie, Institut für Biophysikalische Chemie, Althanstraße 14, 1090, Wien, Austria; b Laboratoire Bioqual, INATAA, Université Frères Mentouri Constantine1, Route de Ain El-Bey, 25000, Constantine, Algeria; c University of Vienna, Faculty of Chemistry, Mass Spectrometry Center, Währinger Straße 38, A-1090, Vienna, Austria; d University of Vienna, Faculty of Chemistry, Department of Analytical Chemistry, Währinger Straße 38, A-1090, Vienna, Austria

**Keywords:** Browning reactions, Tyrosinase, Heterologous expression, Enzyme characterization, Individual phenolics, Antioxidants

## Abstract

In this study, we investigate the effect of enzymatic browning on the phenolic composition of apricot *in vivo* and *in vitro*. The *in vitro* browning was caused by the recombinant latent apricot polyphenol oxidase (L-*Pa*PPO). Successful heterologous expression of *Pa*PPO in *Escherichia coli* yielded substantial amounts of enzyme containing both copper ions in the catalytic active site. The expressed L-*Pa*PPO was characterized with regard to its molecular mass (56531.3 Da), pH optimum (7.0), activation by SDS, and enzyme kinetics. LC-MS/MS was used to compare the phenolic profiles of brown and non-brown apricots. The browning reactions did significantly decrease total phenolics and antioxidant capacity (measured with DPPH and CUPRAC assays). Catechin, epicatechin, and B-type procyanidins were the individual phenolics most affected by browning, followed by chlorogenic and neochlorogenic acid. These phenolics are most likely the main endogenous substrates of L-*Pa*PPO, as they were oxidized much faster than the other identified phenolics.

## Highlights

• Enzymatic browning greatly reduces the amount of phenols in apricots.
• This decrease in phenols causes a marked reduction of the antioxidant capacity.
• Apricot phenols do not contribute evenly to enzymatic browning.
• Catechins and (neo)chlorogenic acids are the main endogenous substrates of *Pa*PPO.

## Graphical abstract

**Figure:**
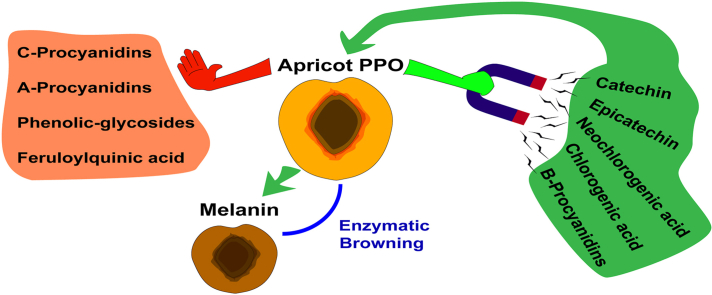

## 1 Introduction

Browning reactions in fruits and vegetables influence the flavor and the quality during storage and processing. The reactions produce undesirable darkening in many foods of plant origin and significantly decrease their functional, organoleptic, and nutritional properties (Vámos-Vigyázó, 1981). In plants, polyphenol oxidase (PPO) is the principal enzyme responsible for browning reactions (Yoruk and Marshall, 2003; Mayer, 2006). PPOs are type 3 copper-containing enzymes that widely occur in many plants, animals, archaea, bacteria, and fungi. Because of their direct involvement in browning reactions, they have been extensively studied, from basic biochemical profiles to the structural base of their enzymatic activity (Yoruk and Marshall, 2003; Mayer, 2006). The apricot enzyme uses molecular oxygen to catalyze the *ortho*-hydroxylation and subsequent oxidation of monophenols (EC 1.14.18.1, monophenolase or cresolase activity) and the oxidation of *o*-diphenols (EC 1.10.3.1, diphenolase or catechol oxidase activity) to highly reactive *o*-quinones. These *o*-quinones polymerize spontaneously to form complex brown pigments (melanins) (Yoruk and Marshall, 2003).

Apricot is one of the most essential fruits globally as it is widely consumed both fresh and in processed forms such as juices, jams, and dried fruits (Madrau et al., 2009; Roussos et al., 2011). The fruit contains high levels of dietary fibers, minerals, vitamins, and carotenoids and can be considered a very rich source of bioactive phenolic compounds, including catechins, phenylpropanoic acids, procyanidins, and phenolic glycosides (Dragovic-Uzelac et al., 2007; Roussos et al., 2011). These compounds are known for their antioxidant activity and are presumed to be responsible for the numerous beneficial effects derived from the consumption of fruits and vegetables (Pokorny et al., 2001). Phenolic compounds have demonstrated strong *in vitro* and *in vivo* antioxidant activities related to their ability to scavenge free radicals, chelate metals, and stop radical chain reactions (Pokorny et al., 2001; Apak et al., 2004). Furthermore, phenolics consumption has been positively correlated with a reduced risk of various diseases originating from oxidative stress, such as cardiovascular diseases and certain cancer types (Pokorny et al., 2001). In addition, their antioxidant activity is also important for preventing oxidation of other food components such as lipids and vitamins (Pokorny et al., 2001). However, fresh and processed apricots are highly affected by enzymatic browning (Madrau et al., 2009; Derardja et al., 2019). Phenolic compounds are the main substrates of PPO, and they are presumed to be the most affected components by enzymatic browning as they are consumed during the reaction (Yoruk and Marshall, 2003).

Phenolic concentrations do vary quantitatively and qualitatively between species and cultivars (Roussos et al., 2011; Leccese et al., 2012). Different apricot cultivars showed significant quantitative variabilities in phenolic contents; however, only slight qualitative differences were reported. Apricots were found to contain high levels of epicatechin, catechin, chlorogenic acid, neochlorogenic acid, and rutin (Dragovic-Uzelac et al., 2007; Madrau et al., 2009; Roussos et al., 2011). The degree of enzymatic browning is closely related to the phenolic content and depends on the quality of individual phenolics (Lee and Jaworski, 1988; Macheix et al., 1990; Murata et al., 1995). Not all the phenolics that are present in the fruit constitute potential PPO substrates. On the one hand, PPO-specificity toward individual phenolics depends on the nature of the compound (diphenolic substrates are usually preferred over triphenolic and monophenolic substrates) (Yoruk and Marshall, 2003; Derardja et al., 2017; Han et al., 2019), and on the other hand, the enzyme specificity diverges significantly between different sources of PPO (Derardja et al., 2017; Han et al., 2019). In addition, *in vitro* studies have shown that the reaction environment and the form of PPO (latent or active) can also affect PPO preference toward phenolic substrates (Nicolas et al., 1994; Cabanes et al., 2007; Derardja et al., 2017). Most of the studies on fruit PPOs were conducted *in vitro* using phenolics that do not occur in the fruits, such as catechol, 4-methylcatechol, and dopamine, and only a few reports investigated the effect of enzymatic browning on the natural substrates of PPO (Lee and Jaworski, 1988; Murata et al., 1995). The impact of enzymatic browning in total, as well as on individual phenolics, needs to be investigated *in vivo* to determine endogenous PPO substrates.

PPO characterization and *in vitro* experiments require relatively high amounts of pure enzyme, which is difficult to collect from the natural source, as exemplified in previous studies (Derardja et al., 2017, 2019). Furthermore, apricot PPO (*Pa*PPO) was found to self-activate spontaneously during storage (Derardja et al., 2017), which would create the need for several purifications over time for a long-term study. This is difficult to achieve as fresh apricots are not available all over the year, and usual storage protocols do not guarantee the preservation of sample authenticity. Heterologous expression, on the other hand, can provide a stable source of well-characterized enzyme whenever needed and is therefore a very suitable option to study *in vitro* enzymatic browning. Successful heterologous expression of plant PPOs in *Escherichia coli* were reported with high quantities of soluble and active pure recombinant PPO (Kampatsikas et al., 2017, 2019; Pretzler et al., 2017; Panis et al., 2020). The obtained PPOs conserved the original properties of the respective PPO extracted from the natural source (Panis et al., 2020). Apricot carries a gene coding for a PPO precursor polypeptide of 597 amino acids with a calculated molecular weight of 67.1 kDa (Chevalier et al., 1999). The pro-enzyme consists of three domains: a signal peptide (∼11 kDa), the catalytically active domain (∼39 kDa), and the C-terminal domain (∼17 kDa) that shields the enzyme's active site (Derardja et al., 2017). Up to date, only one sequence is reported for apricot PPO (UniProt O81103, *Pa*PPO) (Chevalier et al., 1999). In the fruits, the enzyme is present in its latent form (active domain + C-terminal domain) (Chevalier et al., 1999; Derardja et al., 2017) with 496 amino acids (Asp102 → Ser597) and a calculated molecular weight of 56.2 kDa (Chevalier et al., 1999). *In vivo* enzymatic activity is triggered by proteolytic removal of the C-terminal domain (Kampatsikas et al., 2019) and *in vitro* PPO activity is artificially triggered by fatty acids, acidic pH, detergents such as sodium dodecyl sulfate (SDS), and proteases (Yoruk and Marshall, 2003; Mayer, 2006).

Assessing the effect of PPO on total and individual phenolics *in vivo* is affected by the biochemical complexity of the fruits and the presence of other enzymatic systems such as peroxidases that can contribute to enzymatic browning (Vámos-Vigyázó et al., 1981). Thus, it will be more conclusive to assess the effect *in vivo* along with the impact *in vitro* by provoking enzymatic browning with pure PPO obtained from apricots added to the phenolic extract. Bringing together the availability of pure apricot PPO and phenolic extracts from apricots in different stages of freshness, this report implemented the two-pronged strategy for the first time. Therefore, in this work, we investigate the effect of enzymatic browning on total and individual phenolics of apricot (Bafi. cv) and the antioxidant activity of the phenolic extracts. The research is conducted on both *in vivo* and *in vitro* browning. The *in vitro* browning was provoked with pure recombinant *Pa*PPO (the gene was obtained from the same cultivar and was for the first time heterologously expressed in *E. coli*). The successfully expressed L-*Pa*PPO is characterized for its pH optimum, SDS activation and kinetics parameters. Kinetics were investigated with both classic PPO phenolic substrates and L-*Pa*PPO endogenous phenolic substrates. The effect of browning and the deduction of apricot endogenous substrates were achieved by comparing individual phenolic profiles of non-brown and brown (*in vivo* and *in vitro*) apricot at different stages of browning by LC/MS and HPLC-UV.

## 2 Materials and methods

### 2.1 Plant material, cloning and sequencing of *Pa*PPO

Apricot fruits and leaves (*Prunus armeniaca* L., cultivar Bafi) were collected from field-grown trees (M'sila, Algeria), packaged in cool isothermal bag and transported per plane (as cabin baggage) on the same day to Vienna, and stored at −80 °C. RNA was isolated using the Rapid CTAB method according to Gambino et al. (2008). The gene encoding L-*Pa*PPO was cloned into the pGEX-6P-SG expression vector. The experiments are described in detail in the supplementary material (SM).

### 2.2 Heterologous expression and purification of recombinant L-*Pa*PPO

The expression and the purification of L-*Pa*PPO were performed as described previously for tomato PPO (Kampatsikas et al., 2019) with some modifications. L-*Pa*PPO was expressed in *E. coli* SHuffle® T7 cells (NEB), in modified 2xYT medium. The culture was grown at 18 °C under shaking (185 rpm) for 72 h. Enzyme purification was performed using an ÄKTA fast protein liquid chromatography system (FPLC). The detailed methods are presented in the SM.

### 2.3 Gel electrophoresis

Denaturing SDS-PAGE was performed as described by Laemmli (1970) (for details see SM).

### 2.4 Molecular mass determination by nano-LC-MS/MS

The molecular mass of the L-*Pa*PPO was obtained using a high-resolution Linear Trap Quadrupole (LTQ)-Orbitrap Velos mass spectrometer equipped with a nanospray ion source and coupled to a nano HPLC-system. The experimental details are laid out in the SM.

### 2.5 Copper ion content determination

The copper ion content of the enzyme was measured by the method of Hanna et al. (1988). The purified enzyme (600 *μ*g) was mixed with 50 mM sodium ascorbate, the mixture was brought to 400 *μ*L with 100 mM sodium phosphate buffer (pH 6.0), then 600 *μ*L of a 0.5 g L^−1^ 2,2′-biquinoline solution in glacial acetic acid was added. After 10 min of incubation, the absorbance was measured at 546 nm (*ε* = 6300 M^−1^ cm^−1^), and copper ion content was calculated for blank corrected samples.

### 2.6 Enzymatic activity and effect of pH and SDS

L-*Pa*PPO activity measurements were performed at 25 °C in 200 *μ*L of the assay mixture by spectrophotometrically measuring the accumulation of the colored reaction product using a microplate reader (Infinite M200, Tecan). Enzymatic activity was determined from the slope of the initial linear part of the experimental curves (absorbance vs. time) and expressed as specific activity (U mg^−1^). One unit of enzymatic activity (U) was defined as the amount of enzyme that catalyzed the formation of 1 *μ*mol of quinones per minute (1 U = 1 *μ*mol min^−1^). The effect of pH on L-*Pa*PPO activity was studied using tyramine (15 mM) as a monophenolic substrate and dopamine (5 mM) as a diphenolic substrate (with and without 2 mM SDS) at 480 nm. Different pH values ranging from pH 2.0 to 10.0 (pH 2.0 to 6.5 = 50 mM sodium citrate buffer, pH 7.0 to 10.0 = 50 mM Tris HCl buffer) were tested. Furthermore, the effect of different concentrations of SDS (0.5–100 mM) on L-*Pa*PPO activity was investigated. The enzyme showed the highest activity at pH 7.0 with 2 mM of SDS. Therefore, kinetic measurements were carried out at pH 7.0 using 2 mM SDS as an activator.

### 2.7 Enzyme kinetics

In order to determine the kinetic parameters (*k*_cat_, *K*_m_, *k*_cat_/*K*_m_) for L-*Pa*PPO, the enzymatic activity was measured as explained above using different substrates, namely tyramine, dopamine, *L*-DOPA, catechol, chlorogenic acid, neochlorogenic acid, catechin, epicatechin and procyanidin B2, at various molarities. The molar absorption coefficients (ε_λmax_) of the formed quinones (chromophores) for tyramine, dopamine, *L*-DOPA and catechol have already been reported in a previous study (Muñoz et al., 2006). However, for the other substrates, the appropriate wavelength (*λ*_max_) and the molar absorption coefficients (ε_λmax_) of the formed quinones were measured in 50 mM Tris-HCl buffer (pH 7.0) with NaIO_4_ as the chemical oxidant as described by Muñoz et al. (2006). Thus, different amounts of substrate were oxidized chemically with NaIO_4_ and the formed quinones were measured spectrophotometrically using a Shimadzu UV-1800 spectrophotometer (Shimadzu Deutschland, Duisburg, Germany). Later, the molar extinction coefficients were determined by linear regression. The *K*_m_ and *V*_max_ values were determined by nonlinear regression. The maximal turnover rate (*k*_cat_) was calculated by dividing the total substrate converted per min by total molecules of L-*Pa*PPO in the reaction mixture.

### 2.8 Phenolics extraction

Frozen apricots (at commercial maturity) were randomly selected, pitted, and homogenized with a hand blender while they were still cold (≈0 °C). In order to assess the phenolic compounds of apricot during browning quantitatively and qualitatively, the phenolics were extracted at room temperature (≈25 °C) from the obtained puree at several post-homogenization times. Thus, five samples were extracted (1, directly after homogenization; 2, 5 min after homogenization; 3, 30 min after homogenization; 4, 2 h after homogenization; 5, 12 h after homogenization). The samples were conserved in an open recipient covered with a cheese cloth at room temperature (≈25 °C) to allow browning, except for the non-brown puree, which was extracted immediately after homogenization when it was still cold. Phenolic compounds were extracted from the collected samples according to the method described by Dragovic-Uzelac et al. (2007) (details in the SM).

For better identification of the phenolic compounds involved in the enzymatic browning and oxidized by *Pa*PPO, we provoked an *in vitro* enzymatic browning using the phenolic extract of non-brown puree and the purified enzyme. 5 mL of the phenolic extract was freeze-dried and then dissolved in 4.75 mL of 50 mM Tris HCl buffer (pH 7.0). The browning reaction was started by mixing 0.95 mL of the phenolic solution with 25 *μ*L SDS (80 mM) and 5 *μ*L (50 *μ*g) of pure L-*Pa*PPO. The mixture was incubated at room temperature for 5 min, 30 min, 2 h, and 12 h to allow browning before adding 25 *μ*L of HCl (25% m/v) to stop the reaction. The final solution (1 mL) was used for further analysis.

### 2.9 LC-MS and HPLC-UV analysis of phenolics

LC-MS analyses were performed on two different instruments: an UltiMate 3000 series system HPLC equipped with a VWD detector coupled to a maXis UHR ESI-Qq-TOF mass spectrometer, and an Vanquish Horizon UHPLC system coupled to the ESI source of an LTQ Orbitrap Velos mass spectrometer. In both cases, separation was carried out on an Acclaim 120 C18, 2.1 × 150 mm, 3 μm HPLC column (details in the SM). Phenolic compounds were identified on the basis of their elution order, their accurate mass and isotopic pattern, as well as interpretation of the fragmentation pathway and comparison of the MS/MS spectra with literature data and databases such as *mzcloud* (Table S2). The results were also compared with previous reports on apricot phenolics identification by HPLC and LC/MS (Dragovic-Uzelac et al., 2007; Madrau et al., 2009; Rzeppa et al., 2011).

Separation for quantification of the identified phenolics was accomplished by HPLC-UV/DAD, using an Agilent 1260 Infinity system. Chromatographic separations were carried out on a C18 column (Agilent Prep-C18, 250 mm × 4.6 mm, 5 *μ*m) (details in the SM). Identification of neochlorogenic acid, chlorogenic acid, catechin, epicatechin, procyanidin B2, quercetin-3-*O*-rutinoside and quercetin-3-*O*-glucoside was carried out by comparing retention times and spectral data with authentic standards (Fig. S1). The identification of the remaining phenolics was performed by matching the retention times of the corresponding phenolic peaks of the chromatograms obtained with the Agilent HPLC and the chromatograms obtained with LC/MS identification using the same C18 column and the same composition of solvents and gradient elution conditions of the HPLC-UV/DAD. Quantification of these compounds was calculated relative to the corresponding external standards from the calibration curves (Table S3). When pure standards were not available, the concentration of the phenolics was estimated using the calibration curves of the standards closest in chemical structure (as mg of standards equivalents) (Table S3).

### 2.10 Total phenolics, total flavonoids and total *o*-diphenols

Total phenolics (TP) were measured by the Folin-Ciocalteu assay (Singleton et al., 1999). Total flavonoids (TF) were determined using the AlCl_3_ method (Lamaison and Carnart, 1991). Total *o*-diphenols (TOD) were assessed using the method described by Mateos et al. (2001). For total phenolics and total *o*-diphenol, the results were reported as milligrams of gallic acid equivalents per kilogram of fresh weight (mg GAE kg^−1^ FW). For total flavonoids, data were expressed as milligrams of quercetin equivalents per kilogram of fresh fruit (mg QE kg^−1^ FW).

### 2.11 Antioxidant activity

The antioxidant activity was assessed using DPPH (2,2-diphenyl-1-picrylhydrazyl radical) (Brand-Williams et al., 1995) and CUPRAC (cupric reducing antioxidant capacity) (Apak et al., 2004) tests (for details see SM). The calibration curve was prepared with ascorbic acid, and the antioxidant activity of the extracts was expressed as milligrams of ascorbic acid equivalents per kilogram of fresh fruit (mg AAE kg^−1^ FW).

### 2.12 Statistical analysis

All values were expressed as means ± standard deviation of three analytical replicates. Data were processed by analysis of variance (one-way ANOVA) and statistical significance by Tukey's HSD (honestly significant difference) test. Differences at *p* < 0.05 were considered significant. The Pearson correlation coefficients (*r*) were used to determine relationships between phenolic compounds and antioxidant activities. Statistical analysis was performed using Microsoft XLSTAT 2009.

## 3 Result and discussion

### 3.1 Cloning, heterologous expression and protein purification of *Pa*PPO

**Fig. 1:**
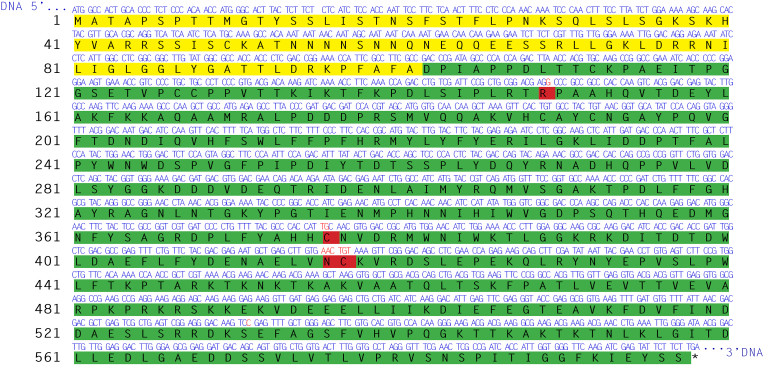
DNA and protein sequences of *Pa*PPO. In blue, DNA sequence (5′➝ 3′); in red, DNA mutation sites. In black, corresponding amino acids (protein sequence); shaded in yellow, signal peptide; shaded in green, latent form; shaded in red, protein mutation sites. (For interpretation of the references to color in this figure legend, the reader is referred to the Web version of this article).

**Fig. 2:**
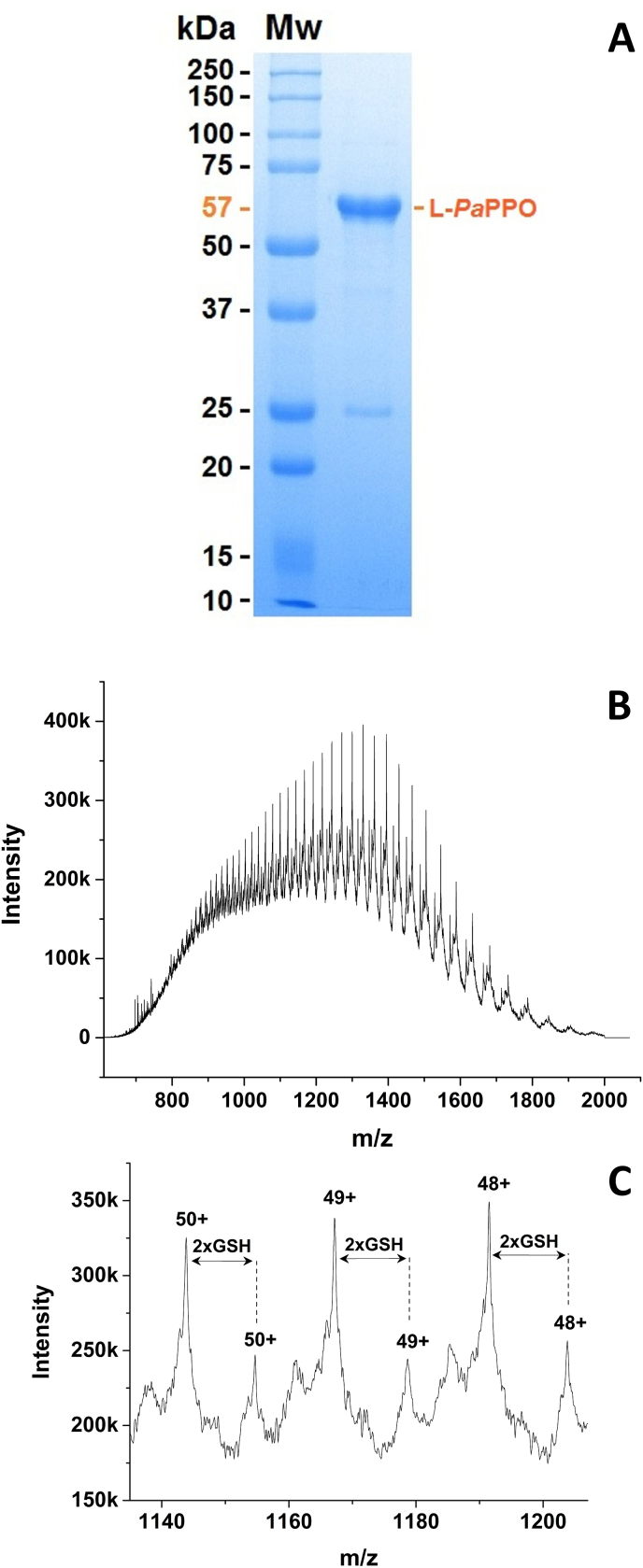
SDS-PAGE and nanoESI mass spectra of the purified recombinant L-*Pa*PPO. **A**, SDS-PAGE gel, (M_w_) molecular weight marker. **B**, entire mass spectrum; **C**, zoomed section of charge states [L-*Pa*PPO + 50H]^50+^ to [L-*Pa*PPO + 48H]^48+^.

Total RNA was successfully isolated from apricot leaves and cDNA was synthesized by reverse transcription. The initial PCR of the gene encoding the L-*Pa*PPO was performed on the *Prunus armeniaca* cDNA with primers enframing the ORF for L-*Pa*PPO (L-*Pa*PPO_*Fw* and L-*Pa*PPO_*Rv*). L-*Pa*PPO (*Pa*PPO without its signal sequence) has an ORF of 1491 bp encoding 496 amino acids, which corresponds to a mass of 56.4 kDa starting with the amino acid sequence DPIAPP. The amplification yielded one band at 1.7 kbp (Fig. S2). By cloning and sequencing, the molecule was identified as the expected gene for L-*Pa*PPO. However, the L-*Pa*PPO (1491bp) gene contained 1721 bp, with an ORF corresponding to only 166 amino acids instead of the expected 496, due to a premature stop codon. By sequencing, this gain in base pairs was discovered to be due to the presence of one 230 bp intron in the gene of L-*Pa*PPO (Table S4). Primers (intron_*Fw* and intron_*Rv*) were designed (Table S1) to remove the intron, which was in turn successfully removed from the L-*Pa*PPO gene (Fig. S2). L-*Pa*PPO is a variant of the published gene encoding *Pa*PPO (UniProt O81103) (Chevalier et al., 1999). The gene was cloned into the expression vector pGEX-6P-SG. The sequencing of the cloned gene (ENA-ID: MZ350792) revealed ten mutations in comparison with the published L-*Pa*PPO sequence (ENA-ID: AF020786), one of them was silent while the others resulted in the exchange of Ser149Arg, Ala376Cys, Arg416Asn and Val417Cys (Fig. 1). The expression vector carrying the L-*Pa*PPO gene was transformed into *E. coli* SHuffle**®** T7 Express cells and the PPO-gene was expressed similar to previous studies (Kampatsikas et al., 2017, 2019; Pretzler et al., 2017). This expression method was very effective for recombinant plant PPOs for high-scale production of pure and active PPO (Kampatsikas et al., 2017, 2019; Pretzler et al., 2017; Panis et al., 2020). The usage of a rich medium (2xYT) in combination with a prolonged expression time (72 h) at 18 °C led to a substantial increase in L-*Pa*PPO yield. Low incubation temperatures around 20 °C were reported to improve the solubility and the stability of recombinant PPO and could increase the number of chaperones in *E. coli* and reduce the activity of proteases that degrade the overexpressed proteins (Sahdev et al., 2008). L-*Pa*PPO was expressed soluble. The expression resulted in a final yield of approximately 45 mg per L of culture. Glutathione-*S*-transferase (GST) is used as a fusion partner to facilitate L-*Pa*PPO purification. The purification was performed using a two-step purification protocol in a pre-packed 5 mL GSTrap FF column (GE). The GST-fusion protein was captured from the total lysate in the first purification step. Later the L-*Pa*PPO was cleaved from the GST-fusion partner by the specific protease HRV 3C, then passed through the column for the second step of purification where only GST-tagged proteins were trapped. The purified L-*Pa*PPO was tested for activity and analyzed by SDS-PAGE to examine the purity and quantity of the proteins (Fig. 2A). The final purity of the enzyme was > 95% as indicated by SDS-PAGE. The purified L-*Pa*PPO (57 kDa) was stored at 4 °C in 200 mM NaCl and 50 mM Tris-HCl pH 7.8 and was used immediately for kinetic measurements and activity assays.

### 3.2 Molecular mass determination

The mass spectra (Fig. 2B and C) of the L-*Pa*PPO revealed the presence of two protein variants with determined masses of 57 141.8 ± 0.5 Da and 56 531.3 ± 0.6 Da, respectively. The protein with the lower mass perfectly matches the calculated molecular weight of 56 531.27 Da for the complete sequence (Fig. S3) of the recombinant L*-Pa*PPO (with 499 amino acids), including the three vector-derived initial amino acids (Gly-Pro-Met) and L-*Pa*PPO from the N-terminal Asp102 to the C-terminal Ser597, with one thioether bridge (-2H) and two disulfide bonds (-4H). The presence of two closed disulfide bonds and one thioether bridge was reported for the enzyme isolated from the natural source (apricot) (Derardja et al., 2017) and it was also reported in many previous heterologous expressions of PPOs (Kampatsikas et al., 2017, 2019; Pretzler et al., 2017; Panis et al., 2020). The mass difference between the two protein variants (57 141.85 and 56 531.28) is 610.57 Da. This gain perfectly fits the mass of two molecules of glutathione (GSH). We presume that the GSH molecules were bound to the remaining two free cysteines introduced by the point mutations (7 cysteines in total: one is part of the thioether bridge located at the active site and four are blocked by the two disulfide bonds) of L-*Pa*PPO during expression or during purification. The presence of GSH binding protein was reported to occur in *E. coli* during heterologous expression (Wang et al., 2018). Lower inducing temperature and IPTG concentration contributed to the soluble expression of glutathione binding proteins (Wang et al., 2018).

### 3.3 Copper ion content determination

The determination of the copper ion content in L-*Pa*PPO was achieved by measuring the absorption (546 nm, *ε* = 6300 M^−1^ cm^−1^) of a Cu(I)-2′-biquinoline complex. The spectrophotometric quantification showed that each molecule of L-*Pa*PPO contains 1.95 ± 0.04 copper ions. Polyphenol oxidases (PPOs) are members of the type-III copper enzyme family that contain a type-III copper center that consists of two copper ions, each coordinated by three conserved histidine residues (Yoruk and Marshall, 2003). Therefore, it seems that almost all the recombinant enzymes contain both copper ions (CuA and CuB) in their active centers.

### 3.4 Biochemical characterization

**Fig. 3:**
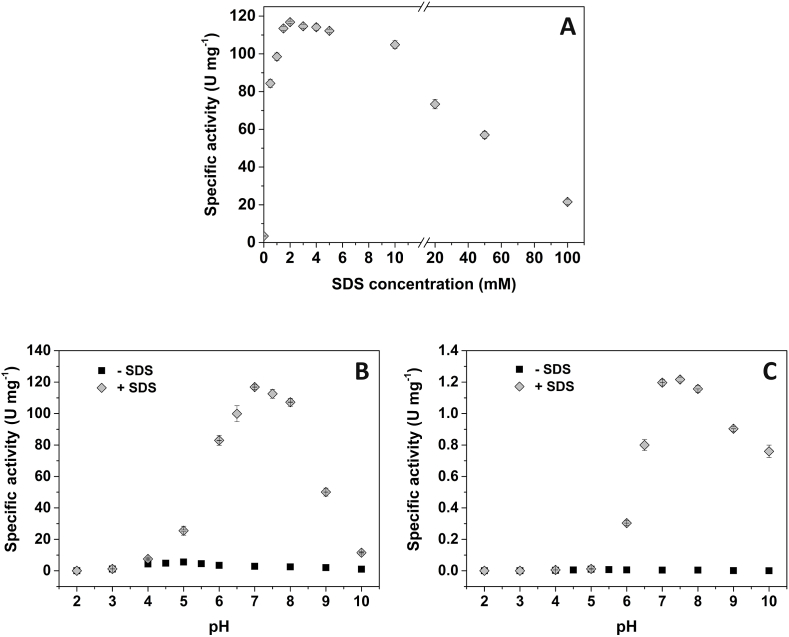
Effect of different concentration of SDS and pH values (2–10) on L-*Pa*PPO activity. **A**, Effect of SDS on the specific activity. **B** and **C** effect of pH: **B**, with dopamine (5 mM) as substrate: **C**, with tyramine (15 mM) as substrate (without SDS, black squares and with 2 mM of SDS, grey diamonds).

Latent PPOs are inactive or partly active, but can be activated by adding detergents such as SDS or fatty acids or by treatment with proteases (Yoruk and Marshall, 2003; Mayer, 2006). Thus, the enzymatic activity of the recombinant L-*Pa*PPO was determined using dopamine as substrate with various concentrations of SDS (0.5–100 mM). As shown in Fig. 3A, *Pa*PPO activity increased rapidly with increasing SDS concentrations until it reached its maximum with 2 mM of SDS. Afterward, *Pa*PPO activity started to diminish gradually with the increase of the SDS concentration, especially with concentrations ≥10 mM. SDS concentrations between 1.5 mM and 6 mM are the best for L-*Pa*PPO activation. Furthermore, the latent enzyme is more active in the presence of 100 mM of SDS than in the absence of SDS. SDS is an activating agent known for its capacity to free PPO from its latent state. SDS activates latent PPOs through conformational changes affected in the enzyme structure (Moore and Flurkey, 1990; Yoruk and Marshall, 2003). However, SDS activation does not follow a linear pattern and the concentration of SDS needed for full activation depends on the kind of PPO, and beyond a certain concentration of the detergent, PPO activity starts to decrease, where high concentrations of SDS can switch the effect from activating to inhibiting (Moore and Flurkey, 1990).

L-*Pa*PPO activity was measured at different pH values, ranging from 2 to 10 using dopamine as the diphenolic substrate (Fig. 3B) and tyramine as the monophenolic substrate (Fig. 3C), both in the presence and in the absence of 2 mM of SDS as an activator. With dopamine, L-*Pa*PPO showed activity in a wide interval of pH (2.0–10.0), with the maximum activity being exhibited at pH 5.0 and 7.0 in the absence and the presence of SDS, respectively. These values are in the range of the pH optimum (4.0–8.0) of plant PPOs (Yoruk and Marshall, 2003). The determination of the pH optimum in the presence and the absence of SDS is necessary, as latent PPO usually exhibits different pH optima at the two conditions. The exhibition of optimal activity at low pH in the absence of SDS is attributed to the acidic activation of the latent form of PPO (Cabanes et al., 2007). The L-*Pa*PPO studied herein was activated *in vitro* by both the acidic environment and SDS. However, the two modes of activation differ considerably in their effectiveness, as the optimal activity of L-*Pa*PPO in the presence of SDS was 24 times higher than at acidic pH in the absence of SDS. Recombinantly expressed apple PPO showed similar pH optima at pH 5.0 in the absence of SDS and at pH 7.0 when activated by SDS. The shift in pH was also attributed to the activation of the latent form in an acidic environment between pH 4.0 and 5.0 (Kampatsikas et al., 2017). Furthermore, the pH optimum of the recombinant enzyme in the absence of SDS is comparable with the pH optimum (4.5 with catechol as substrate) of L-*Pa*PPO extracted from the natural source (Derardja et al., 2017). PPOs usually exhibit a low pH optimum in the absence of detergent and after adding SDS, the optimum pH shifts to a higher value (Cabanes et al., 2007; Kampatsikas et al., 2017). On the other hand, L-*Pa*PPO activity was very weak with tyramine as substrate, where practically no activity was reported in the absence of SDS, and only low activity (100 times lower than with dopamine) was exhibited in the presence of SDS, between pH 6.0 and 10.0 with optimal pH similar to that with dopamine (pH 7.0). This is a common observation for plant PPOs, which often either completely lack monophenolase activity or exhibit only weak activity toward monophenolic substrates (Yoruk and Marshall, 2003; Derardja et al., 2017; Han et al., 2019).

### 3.5 Enzyme kinetics

Kinetic constants of the recombinant L-*Pa*PPO were estimated for nine different substrates (Table 1), including monophenolic (tyramine) and diphenolic substrates (catechol, dopamine, and *L*-DOPA) as well as some phenolic compounds that were identified to be present in the fruit (chlorogenic acid, neochlorogenic acid, catechin, epicatechin and procyanidin B2). Recombinant *Pa*PPO shows higher activity towards most of the phenolic substrates that are present in the natural source in comparison to classic diphenolic and monophenolic substrates used in standard assays. The highest affinity and activity was measured for catechin (*K*_m_ = 1.21 mM, *k*_cat_ = 301 s^−1^) and its isomer epicatechin (*K*_m_ = 1.34 mM, *k*_cat_ = 292 s^−1^) followed by the two isomers neochlorogenic acid (*K*_m_ = 1.47 mM, *k*_cat_ = 268 s^−1^) and chlorogenic acid (*K*_m_ = 1.52 mM, *k*_cat_ = 217 s^−1^). L-*Pa*PPO seems to be more active with these phenolics than with dopamine (*K*_m_ = 1.61 mM, *k*_cat_ = 162 s^−1^) and catechol (*K*_m_ = 3.29 mM, *k*_cat_ = 164 s^−1^). Furthermore, L-*Pa*PPO showed relatively high activity with procyanidin B2, despite that it’s a dimeric compound. The lowest activity was reported with the monophenolic substrate tyramine (*K*_m_ = 4.48 mM, *k*_cat_ = 1.4 s^−1^). Catechin, epicatechin, and chlorogenic acid have been reported as substrates with high efficiency for plant PPOs (Liu et al., 2010; Nicolas et al., 1994), and in many cases, the enzyme exhibited higher affinity towards these compounds than with classic substrates such as catechol (Nicolas et al., 1994). The recombinant L-*Pa*PPO (Bafi. cv) seems to display higher affinities toward catechol, chlorogenic acid, and tyramine in comparison with the previously characterized L-*Pa*PPO (Bulida. cv) (Derardja et al., 2017). The two L-*Pa*PPO are from different cultivars, distinct by 4 amino acid substitutions (Fig. 1). This may have caused the difference as substrate specificity and affinity of PPO was reported to be also dependent on cultivars (Yoruk and Marshall, 2003).

**Table 1:** Kinetic parameters of purified recombinant L-*Pa*PPO.

Substrate	*λ*_max_ (nm)	ε_max_ (M^−1^ cm^−1^)	*k*_cat_ (s^−1^)	*K*_m_ (mM)	*k*_cat_/*K*_m_ (s^−1^ mM^−1^)
Tyramine	480	3300	1.42 ± 0.13	4.48 ± 0.36	0.318 ± 0.029
Dopamine			161.9 ± 3.3	1.61 ± 0.16	99.8 ± 2.9
Catechol	410	1623	163.8 ± 4.1	3.29 ± 0.24	48.4 ± 1.9
*L*-DOPA	475	3600	127.0 ± 4.9	7.16 ± 0.74	17.5 ± 1.7
Chlorogenic acid	400	1700	216.5 ± 4.8	1.52 ± 0.10	141.2 ± 4.1
Neochlorogenic acid			267.5 ± 2.7	1.47 ± 0.29	181.0 ± 2.5
Catechin	440	4850	300.6 ± 5.4	1.215 ± 0.064	246.3 ± 4.1
Epicatechin			291.7 ± 5.9	1.343 ± 0.082	218.5 ± 4.8
Procyanidin B2	390	3780	103.8 ± 2.0	13.32 ± 1.47	7.79 ± 0.80

### 3.6 Effect of browning on total phenolics

The effect of browning on total phenolics of apricot was assessed *in vitro* and *in vivo*, in addition, the antioxidant activity of the phenolic extracts (Table 2) was determined. In this study, apricots were found to be rich in phenolic compounds. The total phenolics content (TP) for the analyzed non-brown apricots (NBA) are shown in Table 2. Results are depicted as mg of gallic acid equivalents (GAE) per kg of fresh apricot (mg GAE kg^−1^ FW). NBA displayed a TP of 669 mg GAE kg^−1^ FW. This value falls in the range of TP (220–1580 mg GAE kg^−1^ FW) reported for apricot by Leccese et al. (2012), but is higher than the range (241–434 mg TAE kg^−1^ FW) reported by Roussos et al. (2011). For the assessment of the nature of the phenolics occurring in apricot, total flavonoids (TF) and total *o*-diphenols (TOD) contents were also investigated. The concentrations are expressed as mg of quercetin and gallic acid equivalents per kg of fresh apricot, respectively. The levels of TF and TOD are presented in Table 2. The values are consistent with the ranges of TF (78.5–127.4 mg kg^−1^ FW) and TOD (137–304.1 mg kg^−1^ FW) reported for apricot by Roussos et al. (2011). TP, TF, and TOD concentrations exhibited significant changes during browning with considerable decreases of all three values. This reduction is expected as phenolic compounds are the main substrates for the enzymes responsible for browning. TP, TF, and TOD contents were considerably affected by *in vitro* browning, with a decrease of 52.5, 73.5, and 77%, respectively, after 12 h of browning. The damage *in vivo* was even more pronounced as a loss of 82.5, 89, and 82% was detected, respectively. This difference is expected as the two types of browning occur under different conditions. The complexity of the *in vivo* browning and the presence of other enzymes (mainly peroxidase) that can also oxidize phenolic compounds (Vámos-Vigyázó et al., 1981) may have emphasized phenolics depletion in comparison to *in vitro* browning. More importantly, the final products of the reaction during *in vivo* browning (melanins) are not extracted with the phenolic compounds due to their low solubility (Huang et al., 2011). However, they are present in the samples of *in vitro* browning. Like most spectrophotometric quantification methods, the two methods of assaying TP and TF are not very specific. In the case of TP and TF, the Folin-Ciocalteu method and the AlCl_3_ method are based on reacting with the hydroxyl groups of the phenolic compounds (Lamaison and Carnart, 1991; Singleton et al., 1999). Thus, melanins may have contributed to the relatively higher content of TP and TF of the *in vitro* BA extracts.

**Table 2:** Effect of browning on phenolics and antioxidant activity.

	NBA	BA				
Phenolics			5 min	30 min	2 h	12 h
TP^1^	668.6 ± 14.5^g^	*in vivo*	439.7 ± 13.3^e^	304.1 ± 10.4^c^	206.5 ± 21.6^b^	118.1 ± 8.7^a^
		*in vitro*	569.2 ± 17.8^f^	448.2 ± 17.5^e^	371.5 ± 19.3^d^	318.6 ± 12.6^c^
TF^2^	109.0 ± 7.1^g^	*in vivo*	46.9 ± 4.9^e^	23.4 ± 4.6^abc^	17.8 ± 1.7^ab^	12.1 ± 1.4^a^
		*in vitro*	76.8 ± 6.0^f^	40.1 ± 2.2^de^	34.8 ± 3.5^cd^	28.8 ± 2.8^bcd^
TOD^1^	271.3 ± 8.3^f^	*in vivo*	139.8 ± 11.7^d^	76.3 ± 4.7^b^	49.8 ± 1.8^a^	48.3 ± 4.3^a^
		*in vitro*	167.2 ± 9.4^e^	136.4 ± 6.8^d^	103.6 ± 4.6^c^	62.1 ± 4.7^ab^
Chlorogenic acid	27.24 ± 0.42^f^	*in vivo*	8.75 ± 0.26^cd^	7.28 ± 0.62^bc^	6.12 ± 0.4^b^	2.74 ± 0.26^a^
		*in vitro*	11.89 ± 1.06^e^	10.18 ± 0.78^de^	7.09 ± 0.57^bc^	5.78 ± 0.83^b^
Neochlorogenic acid	82.98 ± 4.04^f^	*in vivo*	39.69 ± 3.95^e^	32.00 ± 2.64^cd^	26.34 ± 1.19^bc^	8.89 ± 0.68^a^
		*in vitro*	36.88 ± 1.40^de^	34.29 ± 1.04^de^	24.83 ± 2.32^b^	20.82 ± 1.93^b^
(+)-Catechin	15.14 ± 1.62^c^	*in vivo*	3.96 ± 1.04^b^	0.84 ± 0.10^a^	0.00 ± 0.00^a^	0.00 ± 0.00^a^
		*in vitro*	0.54 ± 0.13^a^	0.00 ± 0.00^a^	0.00 ± 0.00^a^	0.00 ± 0.00^a^
(─)-Epicatechin	8.05 ± 1.13^c^	*in vivo*	2.42 ± 0.60^b^	0.00 ± 0.00^a^	0.00 ± 0.00^a^	0.00 ± 0.00^a^
		*in vitro*	0.61 ± 0.02^a^	0.00 ± 0.00^a^	0.00 ± 0.00^a^	0.00 ± 0.00^a^
Procyanidin B1	11.05 ± 1.15^d^	*in vivo*	3.75 ± 0.73^b^	0.43 ± 0.07^a^	0.00 ± 0.00^a^	0.00 ± 0.00^a^
		*in vitro*	5.30 ± 0.62^c^	1.39 ± 0.15^a^	0.89 ± 0.12^a^	0.00 ± 0.00^a^
Procyanidin B2	3.48 ± 0.57^d^	*in vivo*	1.32 ± 0.19^c^	0.71 ± 0.09^b^	0.13 ± 0.02^ab^	0.00 ± 0.00^a^
		*in vitro*	0.44 ± 0.09^b^	0.15 ± 0.03^ab^	0.00 ± 0.00^a^	0.00 ± 0.00^a^
Procyanidin A2	5.63 ± 0.34^e^	*in vivo*	5.00 ± 0.18^d^	4.54 ± 0.20^c^	4.22 ± 0.13^c^	3.69 ± 0.12^b^
		*in vitro*	1.01 ± 0.07^a^	0.97 ± 0.03^a^	0.92 ± 0.03^a^	0.83 ± 0.04^a^
Neochlorogenic acid methyl ester	7.92 ± 0.19^g^	*in vivo*	5.56 ± 0.26^f^	3.92 ± 0.13^e^	2.55 ± 0.21^d^	1.33 ± 0.06^ab^
		*in vitro*	2.04 ± 0.10^c^	1.60 ± 0.11^b^	1.15 ± 0.05^a^	1.12 ± 0.02^a^
3-Feruloylquinic acid	3.61 ± 0.14^c^	*in vivo*	3.23 ± 0.10^bc^	2.64 ± 0.14^a^	2.55 ± 0.14^a^	2.44 ± 0.13^a^
		*in vitro*	3.11 ± 0.09^b^	2.57 ± 0.15^a^	2.54 ± 0.04^a^	2.63 ± 0.18^a^
Quercetin-3-*O*-rutinoside	25.16 ± 0.80^f^	*in vivo*	25.05 ± 0.66^f^	17.04 ± 0.64^e^	13.22 ± 0.70^d^	11.52 ± 0.35^c^
		*in vitro*	9.26 ± 0.22^b^	8.48 ± 0.11^b^	5.85 ± 0.21^a^	5.67 ± 0.19^a^
Quercetin-3-*O*-glucoside	2.83 ± 0.14^e^	*in vivo*	2.45 ± 0.17^d^	2.36 ± 0.13^d^	2.24 ± 0.08^cd^	2.08 ± 0.06^c^
		*in vitro*	1.22 ± 0.03^b^	1.18 ± 0.03^b^	0.89 ± 0.06^a^	1.04 ± 0.05^ab^
Quercetin-3-*O*-(6″-acetyl-glucoside)	2.67 ± 0.07^f^	*in vivo*	2.54 ± 0.08^ef^	2.46 ± 0.04^ef^	2.40 ± 0.06^ed^	2.23 ± 0.07^d^
		*in vitro*	1.94 ± 0.08^c^	1.64 ± 0.12^b^	1.32 ± 0.09^a^	1.17 ± 0.05^a^
Kaempferol-3-*O*-rutinoside	2.34 ± 0.08^c^	*in vivo*	2.33 ± 0.09^c^	2.31 ± 0.13^c^	2.20 ± 0.06^c^	2.17 ± 0.14^c^
		*in vitro*	2.11 ± 0.16^bc^	1.83 ± 0.04^ab^	1.69 ± 0.07^a^	1.58 ± 0.05^a^
DPPH^3^	420.2 ± 16.7^e^	*in vivo*	340.5 ± 13.8^d^	293.4 ± 12.2^c^	195.2 ± 7.0^b^	121.3 ± 9.1^a^
		*in vitro*	445.7 ± 25.2^e^	435.2 ± 16.1^e^	300.1 ± 11.8^cd^	312.1 ± 7.0^cd^
CUPRAC^3^	787.6 ± 20.3^g^	*in vivo*	437.8 ± 12.6^d^	327.8 ± 13.5^c^	153.4 ± 5.5^b^	49.7 ± 3.5^a^
		*in vitro*	642.4 ± 23.3^f^	617.5 ± 15.9^f^	496.2 ± 20.4^e^	453.7 ± 14.4^de^

- NBA, non-brown apricot; BA, brown apricot; TP, total phenolics; TF, total flavonoids; TOD, total *o*-diphenols.

- Values are means ± SD (n = 3), and they are given as mg kg^−1^ of fresh apricot: ^1^ Quantified as gallic acid equivalents, ^2^ Quantified as quercetin equivalents, ^3^ Quantified as ascorbic acid equivalents.

- Significance testing among the different samples was performed by one-way ANOVA followed by Tukey's HSD test. Different superscript letters between columns for the same compound represent significant differences between samples (*p* < 0.05).

### 3.7 Effect of browning on individual phenolics

**Fig. 4:**
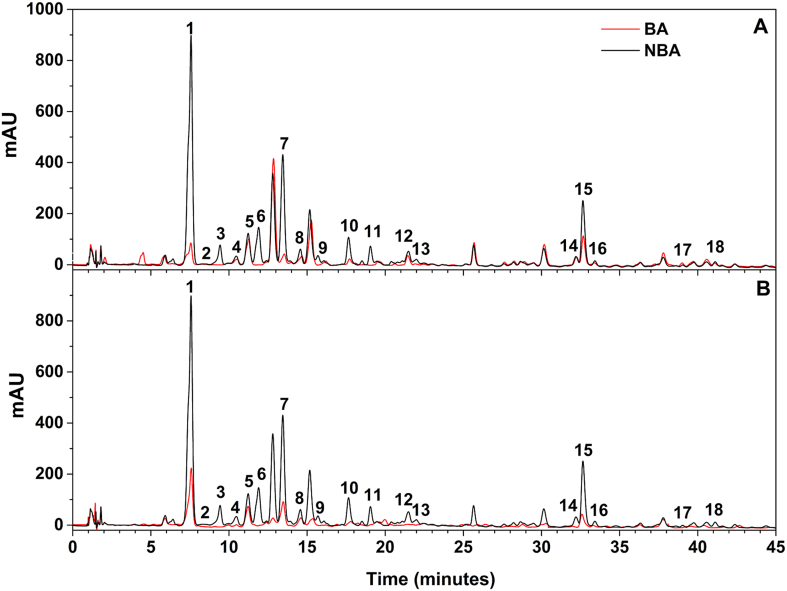
The effect of browning (12 h) *in vivo* (A) and *in vitro* (B) on the phenolic content of apricot fruits cv. ‘‘Bafi’’. NBA, Non-brown apricot; BA, Brown apricot (12 h). Peak identification: 1, Neochlorogenic acid; 2, Procyanidin B3; 3, Procyanidin B1; 4, Procyanidin A2; 5, Procyanidin C2; 6, Catechin; 7, Chlorogenic acid; 8, 3-Feruloylquinic acid; 9, Procyanidin B2; 10, Neochlorogenic acid methyl ester; 11, Epicatechin; 12, Procyanidin C1; 13, Procyanidin B7; 14, Procyanidin B5; 15, Quercetin-3-*O*-rutinoside (Rutin); 16, Quercetin-3-*O*-glucoside; 17, Kaempferol-3-*O*-rutinoside (Nicotiflorin); 18, Quercetin 3-*O*-(6″-acetyl-glucoside). Detection at 278 nm. (For interpretation of the references to color in this figure legend, the reader is referred to the Web version of this article.)

The profiles of individual phenolics obtained with HPLC analyses of NBA and BA (*in vivo* and *in vitro*) are shown in Fig. 4, and the evolution of their amounts at different times of browning are shown in Table 2. Eighteen phenolic compounds were tentatively identified in the chromatograms by LC-MS with eight of them unambiguously confirmed by comparison to reference standards. It can be seen that the major phenolic compounds found in apricots are neochlorogenic acid, chlorogenic acid, quercetin-3-*O*-rutinoside and catechin. Procyanidins were also found to be abundant in apricot, mainly B-type procyanidin (B1, B2, B3, B5, and B7), C-type procyanidin (C1 and C2), and A-type procyanidin (A2) were detected. Other compounds detected were epicatechin, quercetin-3-*O*-glucoside, quercetin-3-*O*-(6″-acetyl-glucoside), kaempferol-3-*O*-rutinoside, neochlorogenic acid methyl ester and 3-feruloylquinic acid. In general, these results are consistent, both quantitatively and qualitatively, with previous reports (Dragovic-Uzelac et al., 2007; Madrau et al., 2009; Roussos et al., 2011) on apricot, where catechin, epicatechin, neochlorogenic acid, chlorogenic acid, rutin and procyanidins were indicated as the main phenolic compounds in apricot (Dragovic-Uzelac et al., 2007; Roussos et al., 2011). However, in our study, we did not detect gallic, caffeic, *p*-coumaric and ferulic acid as reported by some authors (Dragovic-Uzelac et al., 2007). The phenolic profile changes qualitatively and quantitatively between cultivars and during ripening: epicatechin and catechin are known to increase during the progressive growth of the fruit while phenolic acids such as gallic acid and *p*-coumaric acid tend to decrease during fruit ripening (Macheix et al., 1990; Nicolas et al., 1994; Dragovic-Uzelac et al., 2007). Dragovic-Uzelac et al. (2007) reported low amounts of *p*-coumaric and ferulic acid, and only traces of gallic acid at commercial maturity compared to semi-mature and immature fruits. Browning strongly affected the phenolic composition of apricot and a significant loss was observed starting from just a few minutes after homogenization, this loss increased with time and encompassed all the individual phenolics. However, some individual compounds were affected much more severely by the browning and decreased rapidly with time in comparison to other phenolics that were more or less stable during browning.

*In vivo* (Fig. 4A) and *in vitro* (Fig. 4B) browning showed almost the same effect on individual phenolics. However, a few differences towards some individual phenolics can be seen (Table 2). While the degradation of neochlorogenic acid and chlorogenic acid is more pronounced *in vivo*, the degradation of catechin, epicatechin, procyanidins and phenolic glycosides was more pronounced *in vitro*. These differences in rate can be attributed to the fact that the two conditions differ in pH and activation mode (*in vitro* we used SDS to activate PPO). The pH of the medium was reported to change PPO affinity towards phenolic compounds (Vámos-Vigyázó, 1981; Yoruk and Marshall, 2003) and SDS significantly increases PPO activity (Moore and Flurkey, 1990). Furthermore, due to their low solubility, a part of catechins, procyanidins, along with quercetin glycosides occur in plants as insoluble or bound phenolics (Shahidi and Yeo, 2016). This may have sequestered them from enzymatic oxidation *in vivo* in comparison to chlorogenic and neochlorogenic acids, which occur mainly in a soluble form (Shahidi and Yeo, 2016). In both conditions (*in vivo* and *in vitro*), it's clear that flavonols (catechin and epicatechin) and their derivatives (B-type procyanidins) are the phenols most affected by browning. Catechin and epicatechin decreased by 74 and 70% *in vivo* and by 96 and 92% *in vitro*, respectively, after only 5 min of homogenization, and they were completely consumed after 30 min of browning. A similar observation can be noticed regarding procyanidins B1 and B2, of which only traces were detected beyond 2 h of browning. In accordance, the LC-MS data show that all B-type procyanidins (dimeric, trimeric and tetrameric) were fully consumed after 12 h of browning. Chlorogenic and neochlorogenic acids were also potential substrates of enzymatic browning as they diminished by 53–68% after 5 min of browning and both acids were reduced by 90% *in vivo*, and by 79 and 75% *in vitro*, respectively, after 12 h of browning. Procyanidin A2 seems to be oxidized very slowly by L-*Pa*PPO. Similarly, with the notable exception of quercetin-3-*O*-rutinoside, the concentration of phenolic glycosides such as kaempferol-3-*O*-rutinoside, quercetin-3-*O*-glucoside, and quercetin 3-*O*-(6″-acetyl-glucoside), as well as 3-feruloylquinic acid, did not change much during browning in both conditions. These results are compatible with the kinetics of the recombinant L-*Pa*PPO, where the enzyme showed the highest activity and affinity towards catechin and epicatechin, followed by neochlorogenic acid, chlorogenic acid and procyanidin B2. Overall, these findings are in agreement with previous studies on grapes by Lee and Jaworski (1988). These researchers provoked *in vitro* browning by exposing the major phenolic compounds of the fruits to PPO individually and they found that monomeric catechins (catechin and epicatechin) and dimeric procyanidins (type B-procyanidins) cause more intense browning compared to other phenolics in grape. Furthermore, epicatechin was found to be the optimal endogenous substrate of litchi (Liu et al., 2010) and apple PPO (Nicolas et al., 1994). Similarly, chlorogenic acid was found to be mostly responsible for browning in potato (Sanchez-Ferrer et al., 1993), and it was also reported as a key substrate in apples (Murata et al., 1995). On the other hand, phenolic glycosides were reported to be very weak substrates for plant PPOs (Macheix et al., 1990), and C-type procyanidins were found to be oxidized weakly by apple PPO (Nicolas et al., 1994). Catechins and cinnamic acid esters such as chlorogenic acid are the most important natural substrates of PPO in fruits and vegetables. PPO activity is influenced strongly by the nature of the side chain, as well as the number and the position of hydroxyl groups on the benzene ring of the phenolic compound. Substrates with two or three adjacent hydroxyl groups are easily oxidized (Macheix et al., 1990). Thus, the best substrates for PPO are not necessarily the most abundant ones in the fruit the enzyme has been extracted from. For example, while fruits are rich in phenolic glycosides, PPO acts very weakly on these compounds.

### 3.8 Effect of browning on antioxidant activity

The results regarding antioxidant activity (expressed as mg AAE kg^−1^ FW) are presented in Table 2. NBA showed antioxidant activities of 420 mg AAE kg^−1^ FW and 788 mg AAE kg^−1^ FW with DPPH and CUPRAC assays, respectively. The antioxidant capacity of the phenolic extracts (measured with DPPH or CUPRAC assays) was significantly affected by browning. The values displayed the same trend as the TP content during browning, and it was proportional to browning severity. These results suggest that the antioxidant capacity of the extract is derived mainly from phenolic compounds, which is expected as they are the main potent antioxidants present in apricot. This was confirmed with Pearson's statistical correlation analysis, which we used to establish correlations between the antioxidant capacity and TP, TF, TOD and major individual phenolics (Table S5). A strong relationship was found between antioxidant capacity (measured by both assays) and TP, TF, and TOD concentrations during browning. The results of the CUPRAC assay were significantly (*r* > 0.93, *p* < 0.05) correlated with TP, TF, and TOD for *in vitro* and *in vivo* browning. However, the DPPH assay exhibited a significant relationship (*r* > 0.85, *p* < 0.05) with TP, TP and TOD contents only *in vivo* and lower correlations (*r* < 0.8, *p* > 0.05) where reported *in vitro*. The same observation was found for individual phenolics, where most phenolic compounds exhibited a strong correlation with antioxidant capacity *in vivo*, mainly with the CUPRAC assay (0.901 ≤ *r* ≤ 0.992), while lower correlations were reported *in vitro* mainly with the DPPH assay (0.639 ≤ *r* ≤ 0.943). Neochlorogenic acid (*r* = 0.980, with CUPRAC) was found as the phenolic compound with the highest correlation with antioxidant capacity *in vivo*. The *in vitro* browning was performed on the phenolic extract, therefore, the low correlation *in vitro* between the DPPH assay and phenolics can be explained by the formation of new compounds (melanins) with moderate antioxidant activity during the browning process. Melanins were reported to exercise high free radical-scavenging activity (Huang et al., 2011) which may explain the relatively high antioxidant activity reported *in vitro* as compared to the value for *in vivo* browning. Unlike the CUPRAC assay, the DPPH method only measures soluble antioxidants (Apak et al., 2007). Thus, insoluble melanin is hardly expected to react with the DPPH radical. Furthermore, with DPPH, larger molecules (such as melanin) may have a lower chance to access the radical compared to small molecules due to steric inaccessibility (Apak et al., 2007). This may explain the lower antioxidant activity measured with the DPPH assay compared to the CUPRAC assay *in vitro* (Table 2). Melanin's presence in the *in vivo* BA extracts is not expected in significant amounts as its solubility is very low (Huang et al., 2011), preventing its extraction along with the phenolic compounds.

## 4 Conclusion

This study contributes with a new approach to investigate enzymatic browning and determine PPO endogenous substrates. L-*Pa*PPO was successfully expressed (heterologously) in *Escherichia coli* and purified with substantial amounts of enzyme that can be used during *in vitro* studies. The change in phenolic profiles (obtained with LC-MS/MS) during browning provides clear prove of the precedential involvement of certain phenolic substrates in enzymatic browning compared to other phenolic compounds. Catechins and their dimeric derivatives (B-type procyanidins) were found to be the main substrates of *Pa*PPO followed by chlorogenic and neochlorogenic acids. However, phenolic glycosides, tetrameric procyanidins, and 3-feruloylquinic acid were found to be weak substrates of *Pa*PPO. Despite their low concentration compared to rutin and chlorogenic acid, catechin and epicatechin are the most preferred substrates for *Pa*PPO (mainly *in vitro*). These findings indicate clearly that apricot phenolics do not contribute evenly to browning and their contribution is mostly related to *Pa*PPO specificity toward phenolic substrates and not to phenolic abundance. In addition, the similarities between the phenolic profiles during browning *in vitro* and *in vivo* emphasize the role of PPO as the major enzyme responsible for browning in the fruit. The action of PPO on phenolics negatively affected the nutritional properties of the fruits by significantly decreasing total phenolics and antioxidant activity. However, the high antioxidant activity reported after *in vitro* browning compared to *in vivo* browning suggests a possible contribution of melanins in antioxidant activity. Our present work provides researchers with relevant information on apricot PPO, whereby a better understanding of enzymatic browning reactions can be achieved by studying PPO in the closest-to-natural biochemical conditions using endogenous substrates that occur naturally in the fruit instead of classic PPO substrates. Furthermore, knowing PPO endogenous substrates can provide better insights to control enzymatic browning in fruits and fruits products.

## CRediT authorship contribution statement

**Ala eddine Derardja:** Conceptualization, Methodology, Formal analysis, Investigation, Data curation, Writing – original draft. **Matthias Pretzler:** Conceptualization, Methodology, Investigation, Writing – review & editing. **Ioannis Kampatsikas:** Methodology, Investigation, Writing – review & editing. **Milena Radovic:** Investigation. **Anna Fabisikova:** Investigation. **Martin Zehl:** Methodology, Investigation, Data curation, Resources, Writing – review & editing. **Malika Barkat:** Conceptualization, Supervision, Funding acquisition. **Annette Rompel:** Conceptualization, Methodology, Resources, Validation, Writing – review & editing, Supervision, Funding acquisition.

## Declaration of competing interest

The authors declare that they have no known competing financial interests or personal relationships that could have appeared to influence the work reported in this paper.

## Abbreviations

PPO: polyphenol oxidase
*Pa*PPO: polyphenol oxidase from *Prunus armeniaca*
BA: brown apricot
NBA: non-brown apricot
TP: total phenolics
TF: total flavonoids
TOD: total *o*-diphenols
GAE: gallic acid equivalents
QE: quercetine quivalents
AAE: ascorbic acid equivalents
DPPH: 2,2-Diphenyl-1-(2,4,6-trinitrophenyl) hydrazin-1-yl
CUPRAC: cupric reducing antioxidant capacity
FW: fresh weight
TAE: tannic acid equivalents
SDS: sodium dodecyl sulfate
FPLC: fast protein liquid chromatography

## Appendix A Supplementary data

Detailed methods: cloning and sequencing of *Pa*PPO, heterologous expression and purification of recombinant L-*Pa*PPO, gel electrophoresis, molecular mass determination by nano-LC-MS/MS, LC-MS and HPLC-UV analysis of phenolics, Phenolics extraction and quantification, antioxidant activity.

Tables: primers used for L-*Pa*PPO amplification and intron removal, LC-MS data of the phenolic extracts, calibration curves and linearity range of HPLC method, intron of the *Pa*PPO-gene, correlation coefficients (*r*) between antioxidant capacity and phenolics.

Figures: HPLC chromatograms of the phenolic standards, PCR of L-*Pa*PPO gene before and after the removal of the intron, sequence of the expressed L-*Pa*PPO.
