# Supplementary material for: Polyphenol oxidase and enzymatic browning in apricot (Prunus armeniaca L.): Effect on phenolic composition and deduction of main substrates
Source: Curr Res Food Sci. 2022 Jan 4;5:196–206. doi: 10.1016/j.crfs.2021.12.015 (PMC8789516; doi:10.1016/j.crfs.2021.12.015)
Supplement: Multimedia component 1 [file mmc1.pdf]

Supplementary material

Polyphenol oxidase and enzymatic browning in apricot (*Prunus armeniaca* L.): effect on phenolic composition and deduction of main substrates.

Ala eddine Derardja,[1,2] Matthias Pretzler,[1] Ioannis Kampatsikas,[1] Milena Radovic,[1] Anna Fabisikova,[3] Martin Zehl,[3,4] Malika Barkat[2] and Annette Rompel[1]*

[1] Universität Wien, Fakultät für Chemie, Institut für Biophysikalische Chemie, Althanstraße 14, 1090 Wien, Austria, www.bpc.univie.ac.at.

[2] Laboratoire Bioqual, INATAA, Université Frères Mentouri Constantine1, Route de Ain El-Bey, 25000 Constantine, Algeria.

[3] University of Vienna, Faculty of Chemistry, Mass Spectrometry Center, Währinger Straße 38, A-1090 Vienna, Austria.

[4] University of Vienna, Faculty of Chemistry, Department of Analytical Chemistry, Währinger Straße 38, A-1090 Vienna, Austria.

* Corresponding author, E-mail: **annette.rompel@univie.ac.at**

Methods

Plant material, cloning and sequencing of *Pa*PPO

The apricot leaves (150 mg) were ground in liquid nitrogen, and the total RNA was isolated using the Rapid CTAB method according to Gambino et al. (2008) with an extraction buffer containing 2% (m/v) CTAB, 2.5% (m/v) PVP, 2 M NaCl, 100 mM Tris-HCl (pH 8.0), 25 mM EDTA (pH 8.0) and 2% (v/v) of β-mercaptoethanol. Subsequently, cDNA was synthesized using a poly-T primer (5'-T_{25}VN-3') in the presence of Moloney Murine Leukemia Virus reverse transcriptase (MMLV-RT). Reverse transcription reactions were performed in a final reaction volume of 20 μL using 5 μg of total RNA, 20 μM of each dNTP, 20 units of the RNasin® RNase Inhibitor (Promega, USA) and 400 U of MMLV-RT (Promega, USA). RNA was denatured by incubation at 72 °C for 3 min, cooled to 42 °C for 2 min followed by chilling on ice for 5 min. Then, the reaction mixture was added and the samples were incubated for 90 min at 42 °C followed by a heat denaturation step of 10 min at 70 °C. The gene coding for the latent *Pa*PPO (L-*Pa*PPO: *Pa*PPO without the signal peptide; residues Asp102–Ser597) was amplified by PCR using the pair of primers L-*Pa*PPO_*Fw* and L-*Pa*PPO_*Rv* (Table S1) with the Q5® High-Fidelity DNA polymerase (NEB, Ipswich, USA). The reaction was carried out in a final volume of 80 μL containing 4 μL of the cDNA preparation, 650 nM of each primer, 1-fold Q5® reaction buffer (contains 2 mM $MgCl_2$), 20 μM of each dNTP and 0.5 units of Q5® DNA polymerase. Cycling was done in a Mastercycler® pro (Eppendorf; Hamburg, Germany) set to the following program: 95 °C for 3 min, followed by 37 cycles of 95 °C for 20 s, 68 °C for 40 s and 72 °C for 3 min 30 s, with a final elongation step of 10 min at 72 °C. The amplicon from the reaction was purified by agarose gel electrophoresis followed by a silica membrane-based DNA recovery step (Wizard® SV Gel and PCR Clean-Up System from Promega; Madison, WI, USA). The amplicon was cloned into the pGEX-6P-SG expression vector (Biundo et al., 2020). Briefly, 1 fmol of the pGEX-6P-SG vector was incubated with 5 fmol of the purified amplicon, 6 mM ATP, 4 units of Esp3I (Thermo Fisher Scientific), and 160 units of T4 DNA ligase (NEB) in a total volume of 4 μL 1 x CutSmart® buffer for 90 min at 30 °C, followed by thermal denaturation for 5 min at 98 °C and plasmid renaturation for 5 min at 72 °C. The obtained plasmid was transformed into chemically competent *E. coli* TOP10 cells (Thermo Fisher Scientific). Transformants were selected

on LB agar plates with 100 mg L^{-1} ampicillin by incubation at 37 °C overnight. The colonies that formed on the plates were tested for the presence of the L-*Pa*PPO by colony PCR with Taq DNA polymerase and the primers C-PCR_*Fw* and C-PCR_*Rv* (Table S1). In addition to the bacterial colony to be tested, the reaction mixture contained 500 nM of each primer, 2 μM of each dNTP and 0.25 units Hot Start Taq DNA polymerase (from NEB) in a final volume of 10 μL of 1xTaq-buffer. PCR was carried out as follows: 5 min at 95 °C, 30 cycles of 30 s at 95 °C, 60 s at 60 °C, 145 s at 72 °C and a final elongation step of 6 min at 72 °C. A positive clone was grown overnight at 37 °C in LB liquid medium (with 100 mg L^{-1} Na-Ampicillin), which was subsequently used for plasmid DNA extraction (Wizard® Plus SV Minipreps DNA Purification System, Promega). The purified plasmid was analyzed in the region of the insert by Sanger sequencing of both DNA strands (Microsynth; Vienna, Austria). Sequencing of the amplicon revealed the presence of one intron in the *Pa*PPO gene. The intron was removed by amplification of plasmid containing the gene for latent L-*Pa*PPO in the presence of two designed primers (Intron_*Fw* and Intron_*Rv*, Table S1). The reaction was carried out in a total volume of 20 μL containing 650 nM of each primer, 10 fmol of the plasmid, 1-fold Q5® reaction buffer (contains 2 mM $MgCl_2$), 20 μM of each dNTP and 0.5 units of Q5® DNA polymerase. PCR was performed as follows: 3 min at 95 °C, 30 cycles of 20 s at 95 °C, 40 s at 68 °C, 3 min 30 s at 72 °C and a final elongation step of 10 min at 72 °C. The obtained linear plasmid (5 fmol) was digested with the *Esp3I* and *DpnI* restriction enzymes (4 units of each; Thermo Fisher, Scientific) and was ligated with 160 units of T4 DNA ligase (NEB) in a final volume of 10 μL of 1 x CutSmart® buffer containing 1.5 mM of ATP. The construct was transformed again into chemically competent *E. coli* TOP10 cells. The sequence and the removal of the intron were confirmed by colony PCR with Taq DNA polymerase and Sanger sequencing of the obtained plasmid after the selection of positive clones as described above.

Heterologous expression and purification of recombinant L-*Pa*PPO

The expression vector pGEX-6P-SG is endowed with an N-terminal GST-tag with a recognition site for the human rhinovirus 3C protease (HRV-3C) between the tag and the L-*Pa*PPO gene. This recognition site allows a controlled dissociation of the two proteins (GST and L-*Pa*PPO). The constructed plasmid was transformed into chemically competent *E. coli* SHuffle® T7 cells (NEB), and the fusion gene (GST-L-

*Pa*PPO) was efficiently over-expressed using the synthetic *tac* promoter of the pGEX-6P-SG vector. Modified 2xYT medium containing 16 g L^{-1} tryptone-peptone, 10 g L^{-1} yeast extract, 10 g L^{-1} NaCl, 5 g L^{-1} NH_4Cl, 0.5 % (v/v) glycerol, 2 mM $MgCl_2$, and 1 mM $CaCl_2$ (pH 7.5) supplemented with ampicillin (100 mg L^{-1}) was used during the expression to grow bacteria. *E. coli* was grown overnight at 37 °C in a small volume (10 mL) of medium. The resulting saturated overnight culture was used for the inoculation of the expression batch (400 mL) which was firstly grown at 37 °C for ~4 hours until an OD_{600} of ~0.6 cm^{-1} then the temperature was reduced to 18 °C before induction. The culture was induced with 0.5 mM $CuSO_4$ and 0.5 mM isopropyl β-D-1-thiogalactopyranoside. The expression culture remained at 18 °C under shaking (185 rpm) for 72 hours. Afterward, the culture was collected by centrifugation at 8000 × g for 30 minutes at 4 °C. The obtained pellet was resuspended in 40 mL of lysis buffer (50 mM Tris-HCl pH 7.5, 200 mM NaCl, 1 mM EDTA, 50 mM sucrose). Then 0.02 g of lysozyme, 1 mM of phenylmethylsulfonyl chloride (PMSF) and 1 mM benzamidine were added and the resulting suspension was incubated under shaking for 10 minutes on ice. Thereafter, the solution was subjected to five cycles of freezing (in liquid nitrogen) and thawing (in a water bath ~25 °C). The final lysate was supplemented with 2 mM $MgCl_2$ and 0.02 g L^{-1} DNaseI and was incubated for 10 minutes at room temperature (~25 °C), then centrifuged at 20000 × g for 15 minutes at 4 °C. The supernatant was filtered (through a 0.45 μm polyethersulfone membrane) before being applied to an ÄKTA fast protein liquid chromatography system (FPLC) for purification. The filtered solution was loaded onto an affinity column (GSTrap FF, 5 ml from GE Healthcare) pre-equilibrated with 50 mM Tris-HCl pH 7.5, 200 mM NaCl as the binding buffer. After flushing out of unbound proteins, the trapped target proteins were eluted with 50 mM Tris-HCl pH 7.5, 200 mM NaCl and 20 mM reduced glutathione. The eluted protein fractions were concentrated and the buffer was exchanged to 50 mM Tris-HCl pH 7.5, 200 mM NaCl by ultrafiltration using a 20 mL Vivaspin device (30 kDa molecular weight cutoff) driven by centrifugal force (3200 × g, 4 °C). Subsequently, the GST-L-*Pa*PPO fusion protein was cleaved (over 48 hours at 4 °C) with GST-HRV3C-protease (produced in-house, Pretzler et al., 2017) at a mass ratio of 1: 50 (protease): (fusion protein). The cleaved protein was then anew loaded onto the GSTrap FF column using the same buffers as for the first chromatographic step, where the L-*Pa*PPO was eluted immediately in the flow-through while the GST protein and the GST-tagged protease were still trapped by the column. The protein concentration

was determined according to the Lambert-Beer law, the absorption of the enzyme solution was measured at 280 nm and the extinction coefficient was calculated by the ExPASy ProtParam tool (Walker, 2005).

Gel Electrophoresis

To evaluate the purity and molecular mass of the recombinant L-*Pa*PPO, denaturing SDS-PAGE was performed as described by Laemmli (1970) in a mini gel apparatus (Mini-PROTEAN Tetra Cell, Bio-Rad). Enzyme solutions were denatured by heating at 99 °C under reducing conditions (Thermomixer comfort, Eppendorf) for 5 min. The samples were electrophoresed in a 5 % stacking gel and 12 % resolving gel of polyacrylamide. Gels were stained with Coomassie Brilliant Blue G-250, and the molecular weight was estimated by comparison to molecular weight markers (Precision Plus Protein Standard Dual Color, Bio-Rad).

Molecular mass determination by nano-LC-MS/MS

The molecular mass of the L-*Pa*PPO was obtained using a high-resolution Linear Trap Quadrupole (LTQ)-Orbitrap Velos mass spectrometer (Thermo Fisher Scientific, Bremen, Germany) equipped with a nanospray ion source (electrospray voltage: 2.1 kV, ion transfer capillary temperature: 300 °C), coupled to a nano HPLC-system (UltiMate 3000, Dionex). Pure L-*Pa*PPO at a concentration of 10 g L^{-1} was used. The sample was first loaded onto a trap column with loading solvent (ACN/H_2O 2:98 v/v + 0.05 % trifluoroacetic acid). Separation was performed on a C4 analytical column (50 cm × 75 µm Accucore C4, 2.6 µm, 150 Å from Thermo Fisher Scientific) at a flow rate of 300 nL min^{-1} using water and acetonitrile, both modified with 0.1 % formic acid, as mobile phase A and B, respectively. The sample components were separated with a gradient from 2 % to 5 % B in 5 min, from 5 % to 10 % B in 1 min, from 10 % to 60 % B in 14 min, from 60 % to 80 % B in 1 min, followed by an isocratic column cleaning (4 min at 80 % B) and re-equilibration step (4 min at 2 % B). High-resolution ESI-MS (HRESIMS) spectra were recorded in positive ion mode in the range *m/z* 400-2000 with an FT resolution of 7500. MagTran was used for deconvoluting the mass spectrum and determining the molecular mass of L-*Pa*PPO (Zhang & Marshall, 1998).

LC-MS and HPLC-UV analysis of phenolics

LC-MS analyses were performed on two different instruments, namely an UltiMate 3000 series system HPLC equipped with a VWD detector (Dionex/Thermo Fisher Scientific, Germering, Germany) that was coupled to a maXis UHR ESI-Qq-TOF mass spectrometer (Bruker Daltonics, Bremen, Germany) and a Vanquish Horizon UHPLC system (Thermo Fisher Scientific, Bremen, Germany) coupled to the ESI source of an LTQ Orbitrap Velos mass spectrometer (Thermo Fisher Scientific). In both cases, separation was carried out on an Acclaim 120 C18, 2.1 x 150 mm, 3 µm HPLC column (Thermo Fisher Scientific) using water and acetonitrile, both modified with 0.1 % formic acid, as mobile phase A and B, respectively. The sample components were separated with a linear gradient from 3 % to 23 % B in 40 min, from 23 % to 95 % B in 24 min, followed by an isocratic column cleaning (6 min at 95 % B) and re-equilibration step (9 min at 3 % B). The flow rate was 0.45 mL/min and the column oven temperature was set to 25 °C.

On the first system, UV chromatograms (190, 254 and 278 nm) and high-resolution MS spectra in positive ion mode in the range *m/z* 50-1900 were recorded. The following ESI ion source settings were applied: capillary voltage: 4.5 kV, nebulizer: 1.2 bar (N_2), dry gas flow: 7.0 L/min (N_2), and dry temperature: 180 °C. The sum formulas of the detected ions were determined using Bruker Compass Data Analysis 4.0 based on the mass accuracy ($\Delta m/z \leq 5$ ppm) and isotopic pattern matching (SmartFormula algorithm). On the LTQ Orbitrap Velos mass spectrometer, high-resolution ESI-MS spectra (electrospray voltage: 3.0 kV, ion transfer capillary temperature: 380 °C) were recorded in positive and negative ion mode in the range *m/z* 100-1500 with an FT resolution of 60.000. Low-resolution MS/MS spectra of the three most intense precursor ions in each MS^1 spectrum were obtained in automated data-dependent acquisition mode using helium as collision gas and the following settings: activation type: CID, isolation width: $\Delta m/z = 3$, normalized collision energy of 35.0, activation Q: 0.250, and activation time: 30 ms.

HPLC-UV/DAD was accomplished using an Agilent 1260 Infinity system equipped with an Agilent 1260 Infinity diode array detector VL (G 1315D), Agilent 1260 Infinity preparative pump (G 1311C), Agilent 1260 Infinity thermostated column compartment (G 1316A), and Agilent 1260 Infinity preparative auto sampler and fraction collector (G 1364C). Chromatographic separations were carried out on a C18 column (Agilent

Prep-C18, 250 mm× 4.6 mm, 5 μm). Detection was performed with the UV-diode array detector by scanning from 200 to 360 nm. The mobile phases used during the separation consisted of water for mobile phase A and acetonitrile: water (25: 72, v/v) for mobile phase B, with 3 % (v/v) acetic acid in both mobile phases. The following gradient consisting of linear steps was used: 0– 20 min, from 100 % A to 60 % A, 40 % B; 20–45 min, from 60 % A, 40 % B to 30 % A, 70 % B. The same chromatographic conditions were also used for an additional LC-MS analysis to allow for a better correlation of the LC-MS and HPLC-UV data.

Phenolics extraction

Phenolic compounds were extracted according to the method described by Dragovic-Uzelac et al. (2007). 50 g of fruit puree were mixed with 50 mL methanol/HCl (100:1, v/v) containing 5 mM of sodium hydrogen sulfite. The mixture was sonicated for 15 minutes then centrifuged at 3200 g. The supernatant was evaporated to dryness under reduced pressure (35–40 °C) and the residue was re-dissolved in 25 mL of water/ethanol (80:20, v/v). The mixture was extracted four times with 25 mL of ethyl acetate. Later, ethyl acetate fractions were combined, dried for one h with anhydrous sodium sulfate, filtered through Whatman-40 filter paper, and finally, the extract was evaporated to dryness under vacuum (35–40 °C). The obtained residue was re-dissolved in 5 mL of methanol/water (50:50, v/v) and filtered through a 0.45 μm filter before injection into the HPLC system.

Total phenolics, total flavonoids and total *o*-diphenols

Total phenolics (TP) were measured by the Folin-Ciocalteu assay (Singleton et al., 1999). Briefly, 100 μL of each extract was diluted with distilled water (3 mL), to which 250 μL of Folin-Ciocalteu reagent were added. The mixture was shaken and allowed to react at room temperature for 3 min, then 1 mL of 15 % (m/v) Na_2CO_3 was added, and the volume was made up to 5.0 mL with H_2O. The solution was incubated at room temperature for 2 h, after that time the absorbance was measured at 750 nm (25 °C).

Total flavonoids (TF) were determined using the $AlCl_3$ method (Lamaison & Carnart, 1991). 1 mL of the phenolic extracts was mixed with equal volumes of a solution of 2 % $AlCl_3{\cdot}6H_2O$ (2 g in 100 mL methanol). The mixture was incubated for 10 min at room temperature, after which absorbance was measured at 367 nm (25 °C).

Total *o*-diphenols (TOD) were assessed using the colorimetric method described by Mateos et al. (2001). 200 μL of each extract was diluted with 1.4 mL of a 1/1 methanol/water (v/v) mixture. The solution was mixed with 400 μL of a 5 % (m/v) $Na_2MoO_4 \cdot 2H_2O$ solution (prepared in 1/1 methanol/water, v/v). The absorbance was measured at 370 nm (25 °C) after 15 min of incubation at room temperature. The *o*-diphenol content was reported as milligrams of gallic acid equivalents (mg GAE kg^{-1} FW). All the assays were carried out in triplicate.

Antioxidant activity

The ability of the extracts to scavenge DPPH free radicals were estimated by the method of Brand-Williams et al. (1995). Briefly, a 100 μL sample of each extract was added to 2.9 mL of 100 μM DPPH solution in 80/20 methanol/water (v/v). The mixture was shaken vigorously and incubated in darkness for 30 minutes at room temperature, and then the absorbance was recorded at 517 nm (25 °C). The control contained all the reaction reagents except the extract.

For assessing cupric ion reducing ability (CUPRAC), the assay described by Apak et al. (2004) was adapted. 1 mL of a $CuCl_2$ solution (0.01 M) was mixed with 1 mL of a neocuproine (7.5 mM) alcoholic (96 % ethanol) solution and 1 mL of 1 M NH_4Ac buffer (pH 7.0), followed by adding 0.1 mL of phenolic extracts and 1 mL of water. The absorbance was measured at 450 nm (25 °C) after 30 min of incubation in the dark at room temperature (25 °C).

Tables

Table S1. Primers used for L-*Pa*PPO amplification and intron removal.

Purpose	Primer	Sequence (5'-3')
cDNA amplification	L-*Pa*PPO_*Fw*	AGCTCGTCTCCAATGGACCCGATAGCCCCACC
	L-*Pa*PPO_*Rv*	AGCTCGTCTCATCCCTCAAGAAGAATACTCGATCTTGAACCCAC
Intron removal	Intron_*Fw*	AGCTCGTCTCCGCTTCCCAATTCCAGACATTTATACTGACAC
	Intron_*Rv*	AGCTCGTCTCAAAGCCTACTGGAGAGTCCCAGTTCC
Check for L-*Pa*PPO presence	C-PCR_*Fw*	GGAAGTTCTGTTCCAGGGG
	C-PCR_*Rv*	ATAGGGGTTCCGCGCACATTTC

Table S2. LC-MS data of the phenolic extracts.

R_t [min] M1[a]	M2[b]	*m/z* [M+H]⁺	Sum formula (proposed)	Δ to calcd [M+H]⁺	*m/z* [M+Na]⁺	*m/z* [M-H]⁻	Proposed structure	References	mzcloud ID
7.0	2.6	867.2112	$C_{45}H_{38}O_{18}$	-2.2 ppm		865.1956	B-type trimeric procyanidin	Ruiz et al., 2005<br>Rzeppa et al., 2011	
8.3	6.5	355.1013	$C_{16}H_{18}O_9$	-2.9 ppm	377.0837	353.0868	Chlorogenic acid isomer	Nagy & Abrankó, 2016	
**9.4**	**7.6**	**355.1020**	$\mathbf{C_{16}H_{18}O_9}$	**-1.1 ppm**	**377.0835**	**353.0866**	**Neochlorogenic acid**[c]	Ruiz et al., 2005<br>Schmitzer et al., 2011<br>Campbell et al., 2013<br>Nagy & Abrankó, 2016	**7783**
12.7	9.5	579.1496	$C_{30}H_{26}O_{12}$	-0.2 ppm		577.1333	Procyanidin B1	Ruiz et al., 2005<br>Rzeppa et al., 2011	
13.1	8.3	579.1489	$C_{30}H_{26}O_{12}$	-1.3 ppm		577.1334	Procyanidin B3	Rzeppa et al., 2011	
**14.2**	**13.2**	**355.1020**	$\mathbf{C_{16}H_{18}O_9}$	**-1.1 ppm**	**377.0836**	**353.0867**	**Chlorogenic acid**	Ruiz et al., 2005<br>Schmitzer et al., 2011<br>Campbell et al., 2013<br>Nagy & Abrankó, 2016	**962**
14.2	12.6	331.1018	$C_{14}H_{18}O_9$	-1.8 ppm	353.0841	329.0869	Phenolic glycoside	Bottone et al., 2019<br>Deshpande & Peterson, 2020	
**14.3**	**11.7**	**291.0861**	$\mathbf{C_{15}H_{14}O_6}$	**-0.7 ppm**		**289.0709**	**Catechin**	Rzeppa et al., 2011<br>Campbell et al., 2013	**1524**
15.1	14.3	369.1174	$C_{17}H_{20}O_9$	-1.8 ppm	391.0993	367.1023	3-Feruloylquinic acid	Nagy & Abrankó, 2016	
16.1	14.8	331.1015	$C_{14}H_{18}O_9$	-2.6 ppm		329.0868	Phenolic glycoside	Bottone et al., 2019<br>Deshpande & Peterson, 2020	
16.2	11.3	867.2114	$C_{45}H_{38}O_{18}$	-2.0 ppm		865.1955	B-type trimeric procyanidin	Ruiz et al., 2005<br>Rzeppa et al., 2011	
16.3	12.0	867.2114	$C_{45}H_{38}O_{18}$	-2.0 ppm		865.1955	B-type trimeric procyanidin	Ruiz et al., 2005<br>Rzeppa et al., 2011	
16.5	12.3	579.1480	$C_{30}H_{26}O_{12}$	-3.0 ppm		577.1335	Procyanidin B4	Ruiz et al., 2005<br>Rzeppa et al., 2011	

16.5	10.6	1155.2743	$C_{60}H_{50}O_{24}$	-1.9 ppm		1153.2578	B-type tetrameric procyanidin	Rzeppa et al., 2011	
17.8	17.3	369.1175	$C_{17}H_{20}O_9$	-1.3 ppm	391.0993	367.1024	Neochlorogenic acid methyl ester	Jaiswal & Kuhnert, 2011<br>Crupi et al., 2018	
**18.0**	**15.3**	**579.1494**	$\mathbf{C_{30}H_{26}O_{12}}$	**-0.5 ppm**		**577.1336**	**Procyanidin B2**	Ruiz et al., 2005<br>Rzeppa et al., 2011	
18.0	13.7	867.2116	$C_{45}H_{38}O_{18}$	-1.7 ppm		865.1960	B-type trimeric procyanidin	Ruiz et al., 2005<br>Rzeppa et al., 2011	
18.6	17.4	331.1020	$C_{14}H_{18}O_9$	-1.1 ppm	353.0840	329.0869	Phenolic glycoside	Bottone et al., 2019<br>Deshpande & Peterson, 2020	
**19.8**	**18.6**	**291.0863**	$\mathbf{C_{15}H_{14}O_6}$	**-0.2 ppm**		**289.0710**	**Epicatechin**	Rzeppa et al., 2011<br>Campbell et al., 2013<br>Jang et al., 2018	**23**
22.4	19.1	867.2110	$C_{45}H_{38}O_{18}$	-2.4 ppm		865.1955	B-type trimeric procyanidin	Ruiz et al., 2005<br>Rzeppa et al., 2011	
22.9	20.7	867.2111	$C_{45}H_{38}O_{18}$	-2.3 ppm		865.1955	B-type trimeric procyanidin	Ruiz et al., 2005<br>Rzeppa et al., 2011	
24.0	21.6	579.1492	$C_{30}H_{26}O_{12}$	-0.8 ppm		577.1335	Procyanidin B7	Rzeppa et al., 2011	
24.9	22.4	867.2110	$C_{45}H_{38}O_{18}$	-2.4 ppm		865.1956	B-type trimeric procyanidin	Ruiz et al., 2005<br>Rzeppa et al., 2011	
25.1	22.7	867.2110	$C_{45}H_{38}O_{18}$	-2.4 ppm		865.1952	B-type trimeric procyanidin	Ruiz et al., 2005<br>Rzeppa et al., 2011	
25.2	26.2	369.1175	$C_{17}H_{20}O_9$	-1.4 ppm	391.0994	367.1022	Chlorogenic acid methyl ester	Jaiswal & Kuhnert, 2011<br>Crupi et al., 2018	
**29.1**	**31.6**	**611.1604**	$\mathbf{C_{27}H_{30}O_{16}}$	**-0.4 ppm**		**609.1433**	**Quercetin-3-*O*-rutinoside (rutin)**	Ruiz et al., 2005<br>Schmitzer et al., 2011<br>Campbell et al., 2013<br>Jang et al., 2018	**28**
**29.9**	**32.2**	**465.1020**	$\mathbf{C_{21}H_{20}O_{12}}$	**-1.6 ppm**		**463.0867**	**Quercetin-3-*O*-glucoside**	Schmitzer et al., 2011<br>Campbell et al., 2013<br>Jang et al., 2018	**1472**
30.2	31.0	579.1487	$C_{30}H_{26}O_{12}$	-1.7 ppm		577.1334	Procyanidin B5	Rzeppa et al., 2011	

32.8	37.4	595.1646	$C_{27}H_{30}O_{15}$	-1.9 ppm	593.1493	Kaempferol-3-*O*-rutinoside (nicotiflorin)	Ruiz et al., 2005<br>Campbell et al., 2013<br>Jang et al., 2018	**8177**
34.7	39.3	507.1122	$C_{23}H_{22}O_{13}$	-2.3 ppm	505.0972	Quercetin-3-*O*-(6"-*O*-acetyl)glucoside	Ruiz et al., 2005<br>Schmitzer et al., 2011<br>Campbell et al., 2013<br>Jang et al., 2018	

[a]Method using the Acclaim 120 C18, 2.1 x 150 mm, 3 μm HPLC column and water + 0.1 % formic acid and acetonitrile + 0.1 % formic acid as mobile phase A and B, respectively.

[b]Method using the Agilent Prep-C18, 4.6×250 mm, 5 μm HPLC column and water + 3 % acetic acid and acetonitrile:water:acetic acid (25:72:3, v/v) as mobile phase A and B, respectively.

[c]Compounds in bold were confirmed by comparison of the retention times and UV spectra with authentic references (see Fig. S1).

Table S3. Calibration curves and linear range of the HPLC method.

Standards	Linearity range (μg/mL)	R^2	Regression equation[a]
Neochlorogenic acid	0.8 – 350	0.9998	y = 27.74x - 28.94
Catechin	1.45 – 290	0.9999	y = 24.48x - 19.77
Chlorogenic acid	0.75 – 354	0.9999	y = 35.19x - 14.53
Procyanidin B2	1.6 – 217	0.9997	y = 16.18x - 10.74
Epicatechin	0.72 – 290	0.9999	y = 18.43x - 23.48
Quercetin-3-*O*-rutinoside	2.4 – 97.5	0.9999	y = 23.16x - 7.357
Quercetin-3-*O*-glucoside	2.9 – 115.5	0.9999	y = 18.43x - 15.28

Procyanidin B2 was used to calculate the concentration of other procyanidins; quantification of quercetin and kaempferol glycosides was performed using the quercetin-3-*O*-rutinoside calibration curve; chlorogenic acid was used to estimate the concentration of neochlorogenic acid methyl ester.

[a] y: amount in µg, x: peak area in mAU min.

Table S4. Intron of the *Pa*PPO-gene.

Sequence (5’-3’)
GTAGGTTTTTAATTTCTTTCTTCGTTATTATTTTACTCTTAATTTTGT<br>ATGGTTAAATTGAGAATGTACACCTTATATTGTCAACATAAACATT<br>TATCTCCGAAAATAAGTAGAAAAATATCCATTTGCCACTGCATTTT<br>TCTGAAAAATTTGTCTCAATACTTTTGTATATTTTCATATGGCTCCT<br>TAAAATTGAGCTCCTCACTCTGACACTTGCTATGTTTCTGTAG

Table S5. Correlation coefficients (*r*) between antioxidant capacity (based on DPPH and CUPRAC assays) and the major phenolic compounds detected.

	DPPH assay				CUPRAC assay			
	in vivo		*in vitro*		*in vivo*		*in vitro*	
Phenolics	*r*	*p*	*r*	*p*	*r*	*p*	*r*	*p*
TP	0.967	0.007	0.775	0.007	0.996	<0.001	0.972	<0.001
TF	0.864	0.059	0.639	0.059	0.962	0.009	0.932	0.009
TOD	0.882	0.048	0.660	0.048	0.969	0.006	0.979	0.006
Chlorogenic acid	0.833	0.079	0.865	0.058	0.942	0.016	0.964	0.008
Neochlorogenic acid	0.916	0.029	0.857	0.063	0.980	0.003	0.958	0.010
Catechin	0.819	0.090	0.709	0.180	0.938	0.018	0.864	0.059
Epicatechin	0.811	0.095	0.727	0.163	0.930	0.022	0.878	0.050
Procyanidin B1	0.836	0.078	0.891	0.042	0.944	0.016	0.968	0.007
Procyanidin A2	0.986	0.002	0.715	0.175	0.983	0.003	0.867	0.057
Neochlorogenic acid methyl ester	0.981	0.003	0.774	0.125	0.992	0.001	0.910	0.032
Quercetin-3-*O*-rutinoside	0.941	0.017	0.702	0.186	0.901	0.037	0.858	0.062
Kaempferol-3-*O*-rutinoside	0.987	0.002	0.943	0.016	0.931	0.022	0.997	<0.001

- TP, total phenolics; TF, total flavonoids; TOD, total *o*-diphenols.
- Statistical relationship was determined with Pearson correlation coefficients (*r*).

Figures

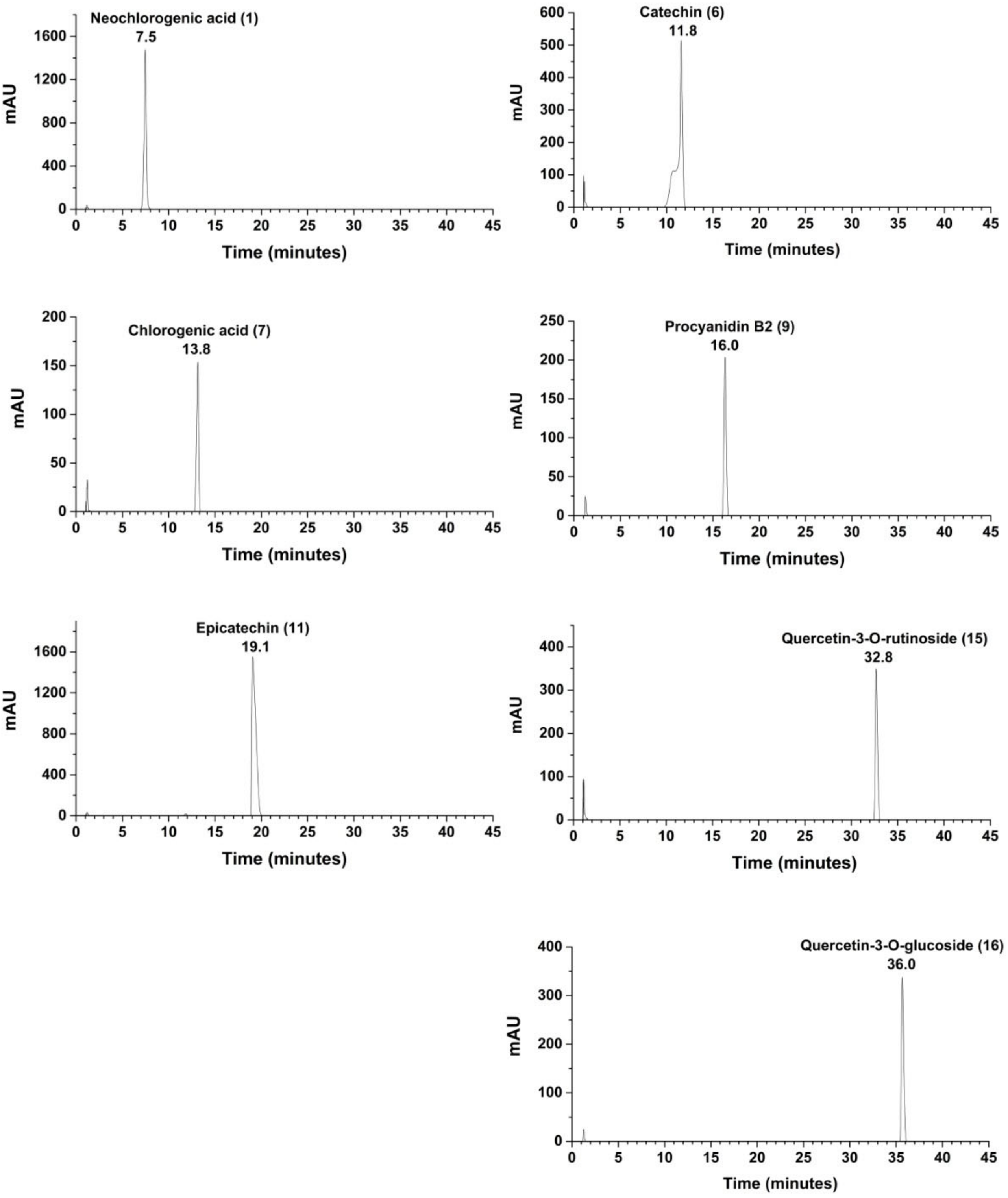

Fig. S1. HPLC chromatograms of the phenolic standards. Injected amounts: Neochlorogenic acid (20 μg), catechin (10 μg), chlorogenic acid (2 μg), procyanidin B2 (6 μg), epicatechin (30 μg), quercetin-3-*O*-rutinoside (5 μg), quercetin-3-*O*-glucoside (6 μg). UV detection at 278 nm.

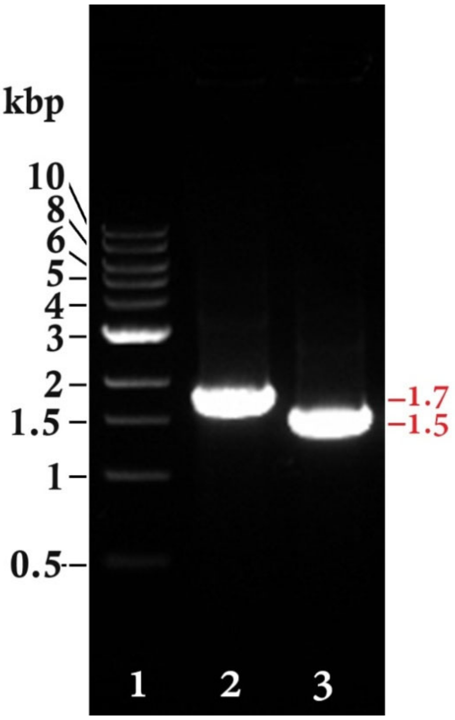

Fig. S2. PCR of the L-*Pa*PPO gene before and after the removal of the intron. Line 1, molecular size standard; Line 2, L-*Pa*PPO gene with its intron; Line 3, L-*Pa*PPO gene after intron removal.

```
 −3  GPM
  1  DPIAPPDLTTCKPAEITPGGSETVPCCPPVTTKIKTFKPDLSIPLRTRPA
 51  AHQVTDEYLAKFKKAQAAMRALPDDDPRSMVQQAKVHCAYCNGAYPQVGF
101  TDNDIQVHFSWLFFPFHRMYLYFYERILGKLIDDPTFALPYWNWDSPVGF
151  PIPDIYTDTSSPLYDQYRNADHQPPVLVDLSYGGKDDDVDEQTRIDENLA
201  IMYRQMVSGAKTPDLFFGHAYRAGNLNTGKYPGTIENMPHNNIHIWVGDP
251  SQTHQEDMGNFYSAGRDPLFYAHHCNVDRMWNIWKTLGGKRKDITDTDWL
301  DAEFLFYDENAELVNCKVRDSLEPEKQLRYNYEPVSLPWLFTKPTARKTK
351  NKTKAKVAATQLTSKFPATLVEVTTVEVARPKPRKRSKKEKVDEEELLII
401  KDIEFEGTEAVKFDVFINDDAESLSRRDKSEFAGSFVHVPQGKTTKAKTK
451  TNLKLGITDLLEDLGAEDDSSVLVTLVPRVSNSPITIGGFKIEYSS*
```

Fig. S3. Sequence of the expressed L-*Pa*PPO. Shaded in red: vector-derived amino acids.

References

Apak, R., Güçlü, K., Özyürek, M., & Karademir, S. E. (2004). Novel total antioxidant capacity index for dietary polyphenols and vitamins C and E, using their cupric ion reducing capability in the presence of neocuproine: CUPRAC method. *Journal of Agricultural and Food Chemistry*, *52*(26), 7970–7981. https://doi.org/10.1021/jf048741x.

Biundo, A., Braunschmid, V., Pretzler, M., Kampatsikas, I., Darnhofer, B., Birner-Gruenberger, R., Rompel, A., Ribitsch, D., & Guebitz, G. M. (2020). Polyphenol oxidases exhibit promiscuous proteolytic activity. *Communications Chemistry*, *3*(1), 62. https://doi.org/10.1038/s42004-020-0305-2.

Bottone, A., Masullo, M., Montoro, P., Pizza, C., & Piacente, S. (2019). HR-LC-ESI-Orbitrap-MS based metabolite profiling of *Prunus dulcis* Mill. (Italian cultivars Toritto and Avola) husks and evaluation of antioxidant activity. *Phytochemical Analysis*, *30* (4), 415–423. https://doi.org/10.1002/pca.2824.

Brand-Williams, W., Cuvelier, M.-E., & Berset, C. (1995). Use of a free radical method to evaluate antioxidant activity. *LWT-Food Science and Technology*, *28*(1), 25–30. https://doi.org/10.1016/S0023-6438(95)80008-5.

Campbell, O. E., Merwin, I. A., & Padilla-Zakour, O. I. (2013). Characterization and the Effect of Maturity at Harvest on the Phenolic and Carotenoid Content of Northeast USA Apricot (*Prunus armeniaca*) Varieties. *Journal of Agricultural and Food Chemistry*, *61* (51), 12700–12710. https://doi.org/10.1021/jf403644r.

Crupi, P., Bleve, G., Tufariello, M., Corbo, F., Clodoveo, M. L., & Tarricone, L. (2018). Comprehensive identification and quantification of chlorogenic acids in sweet cherry by tandem mass spectrometry techniques. *Journal of Food Composition and Analysis*, *73*, 103-111. https://doi.org/10.1016/j.jfca.2018.06.013.

Deshpande, S., & Peterson, D. G. (2020). Identification of Somatosensory Compounds Contributing to Slipperiness and Thickness Perceptions in Canned Prunes (*Prunus domestica*). *Journal of Agricultural and Food Chemistry*, *68* (46), 13160–13167. https://doi.org/10.1021/acs.jafc.0c00544.

Dragovic-Uzelac, V., Levaj, B., Mrkic, V., Bursac, D., & Boras, M. (2007). The content of polyphenols and carotenoids in three apricot cultivars depending on stage of maturity and geographical region. *Food Chemistry*, *102*(3), 966–975. https://doi.org/10.1016/j.foodchem.2006.04.001.

Gambino, G., Perrone, I., & Gribaudo, I. (2008). A rapid and effective method for RNA extraction from different tissues of grapevine and other woody plants. *Phytochemical Analysis*, *19*(6), 520–525. https://doi.org/10.1002/pca.1078.

Jaiswal, R., & Kuhnert, N. (2011). How to identify and discriminate between the methyl quinates of chlorogenic acids by liquid chromatography-tandem mass spectrometry. *Journal of Mass Spectrometry*, *46*(3), 269–281. https://doi.org/10.1002/jms.1889.

Jang, G. H., Kim, H. W., Lee, M. K., Jeong, S. Y., Bak, A. R., Lee, D. J., & Kim, J. B. (2018). Characterization and quantification of flavonoid glycosides in the *Prunus* genus by UPLC-DAD-QTOF/MS. *Saudi Journal of Biological Sciences*, *25*(8), 1622-1631. https://doi.org/10.1016/j.sjbs.2016.08.001.

Laemmli, U. K. (1970). Cleavage of structural proteins during the assembly of the head of bacteriophage T4. *Nature*, *227*(5259), 680. https://doi.org/10.1038/227680a0.

Lamaison, J. L., & Carnart, A. (1991). Teneurs en principaux falvonoïdes des fleurs et des feuilles de *Crataegus monogyna* Jacq. Et de *Crataegus laevigata* (Poiret) DC. en fonction de la période de végétation. *Plantes Médicinales et Phytothérapie*, *25*(1), 12–16.

Mateos, R., Espartero, J. L., Trujillo, M., Rios, J. J., León-Camacho, M., Alcudia, F., & Cert, A. (2001). Determination of phenols, flavones, and lignans in virgin olive oils by solid-phase extraction and high-performance liquid chromatography with diode array ultraviolet detection. *Journal of Agricultural and Food Chemistry*, *49*(5), 2185–2192. https://doi.org/10.1021/jf0013205.

Nagy, Á., & Abrankó, L. (2016). Profiling of hydroxycinnamoylquinic acids in plant extracts using in-source CID fragmentation. *Journal of Mass Spectrometry*, *51*(12), 1130–1145. https://doi.org/10.1002/jms.3847.

Pretzler, M., Bijelic, A., & Rompel, A. (2017). Heterologous expression and characterization of functional mushroom tyrosinase (*Ab*PPO4). *Scientific Reports*, *7*(1), 1810. https://doi.org/10.1038/s41598-017-01813-1.

Ruiz, D., Egea, J., Gil, M. I., & Tomás-Barberán, F. A. (2005). Characterization and quantitation of phenolic compounds in new apricot (*Prunus armeniaca* L.) varieties. *Journal of Agricultural and Food Chemistry*, *53*(24), 9544–9552. https://doi.org/10.1021/jf051539p.

Rzeppa, S., Von Bargen, C., Bittner, K., & Humpf, H.-U. (2011). Analysis of Flavan-3-Ols and Procyanidins in Food Samples by Reversed Phase High-Performance Liquid Chromatography Coupled to Electrospray Ionization Tandem Mass Spectrometry (RP-HPLC-ESI-MS/MS). *Journal of Agricultural and Food Chemistry*, *59* (19), 10594–10603. https://doi.org/10.1021/jf202697j.

Schmitzer, V., Slatnar, A., Mikulic-Petkovsek, M., Veberic, R., Krska, B., & Stampar, F. (2011). Comparative study of primary and secondary metabolites in apricot (*Prunus armeniaca* L.) cultivars. *Journal of the Science of Food and Agriculture*, *91*(5), 860–866. https://doi.org/10.1002/jsfa.4257.

Singleton Vernon, L., Orthofer, R., & Lamuela-Raventos, R. M. (1999). Analysis of total phenols and other oxidation substrates and antioxidants by means of folin-ciocalteu reagent. *Methods Enzymol. C*, *299*, 152–178. https://doi.org/10.1016/S0076-6879(99)99017-1.

Walker, J. M. (2005). *The proteomics protocols handbook*. Springer. https://doi.org/10.1385/1592598900.

Zhang, Z., & Marshall, A. G. (1998). A universal algorithm for fast and automated charge state deconvolution of electrospray mass-to-charge ratio spectra. *Journal of the American Society for Mass Spectrometry*, *9*(3), 225–233. https://doi.org/10.1016/S1044-0305(97)00284-5.
